# Supplementary material for: Site Selectivity in Pd-Catalyzed Reactions of α-Diazo-α-(methoxycarbonyl)acetamides: Effects of Catalysts and Substrate Substitution in the Synthesis of Oxindoles and β-Lactams
Source: Molecules. 2019 Sep 30;24(19):3551. doi: 10.3390/molecules24193551 (PMC6803982; doi:10.3390/molecules24193551)
Supplement: Supplementary file 1 [file molecules-24-03551-s001.pdf]

Site Selectivity in Pd-Catalyzed Reactions of α-Diazo-α-(methoxycarbonyl)acetamides: Effects of Catalysts and Substrate Substitution in the Synthesis of Oxindoles and β-Lactams

D. Solé,*[a] F. Pérez-Janer,[a] A. Amenta,[a] M.-L. Bennasar[a] and I. Fernández*[b]

[a] *Laboratori de Química Orgànica, Facultat de Farmàcia i Ciències de l'Alimentació, Universitat de Barcelona, Av. Joan XXIII 27-31, 08028-Barcelona, Spain, E-mail: dsole@ub.edu.*

[b] *Departamento de Química Orgánica I and Centro de Innovación en Química Avanzada (ORFEO-CINQA), Facultad de Ciencias Químicas, Universidad Complutense de Madrid, 28040-Madrid, Spain, Email: israel@.quim.ucm.es.*

Electronic Supplementary Information

Table of Contents

Copies of ^{1}H and ^{13}C NMR spectra of new compounds **S2-S24**

Cartesian coordinates and total energies **S25-S41**

Copies of ^{1}H and ^{13}C NMR spectra of new compounds

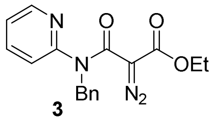

3

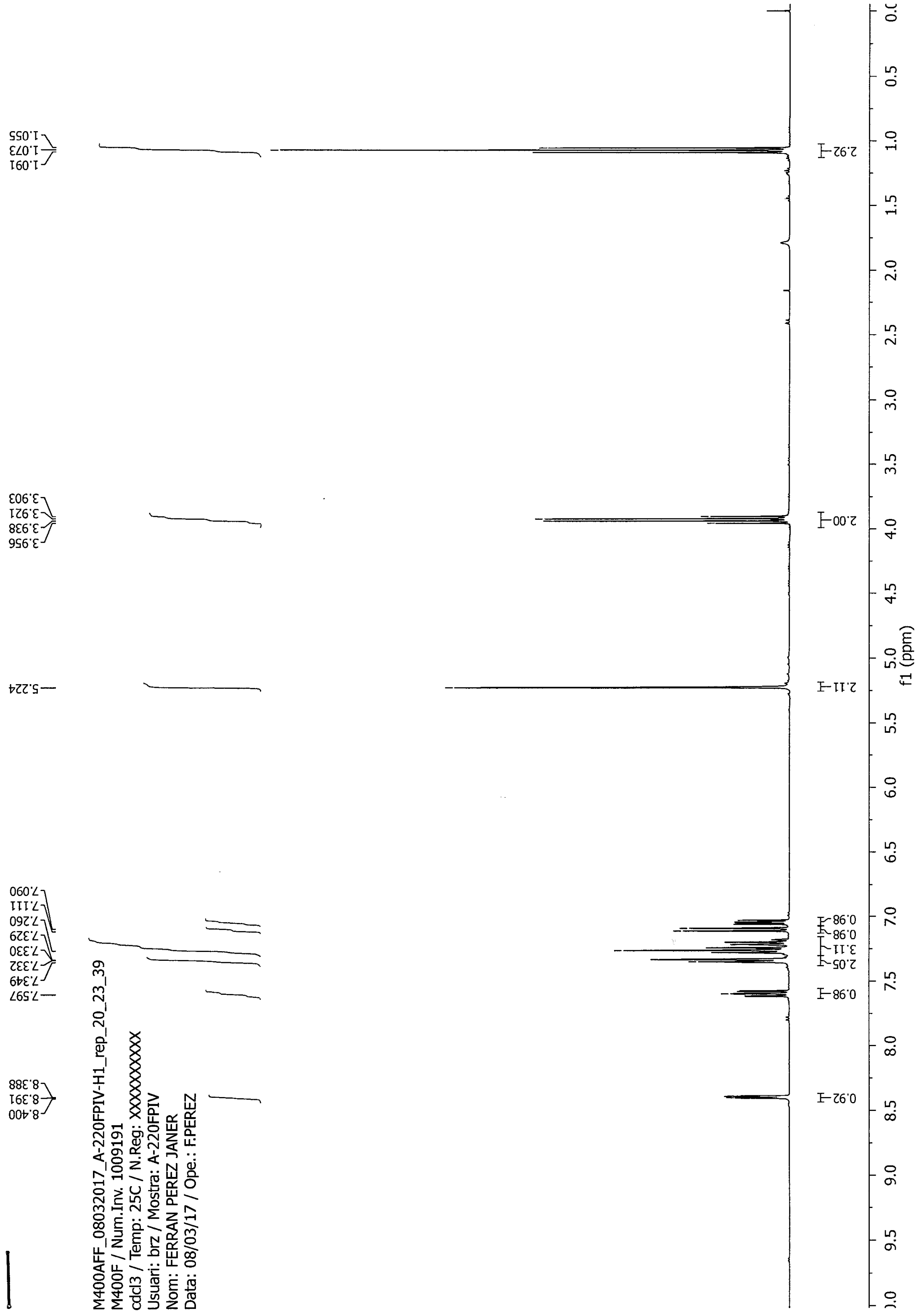

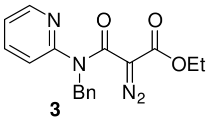
N
O
O
N
OEt
Bn
N2

3

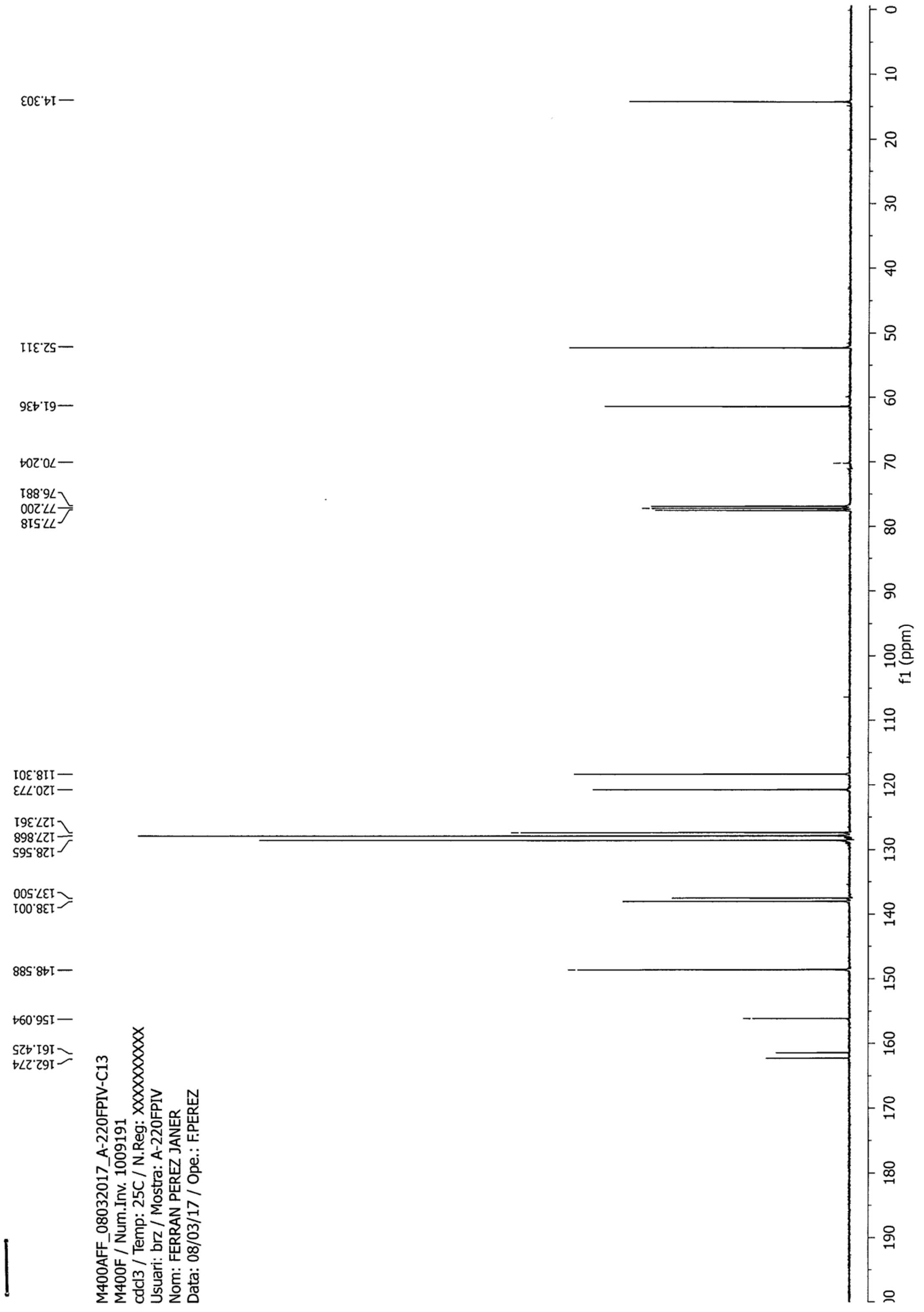
14.303
52.311
61.436
70.204
76.881
77.200
77.518
118.301
120.773
127.361
127.868
128.565
137.500
138.001
148.588
156.094
161.425
162.274
M400AFF_08032017_A-220FPIV-C13
M400F / Num.Inv. 1009191
cdcl3 / Temp: 25C / N.Reg: XXXXXXXXXXX
Usuari: brz / Mostra: A-220FPIV
Nom: FERRAN PEREZ JANER
Data: 08/03/17 / Ope.: F.PEREZ
f1 (ppm)

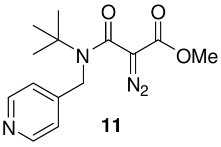

11

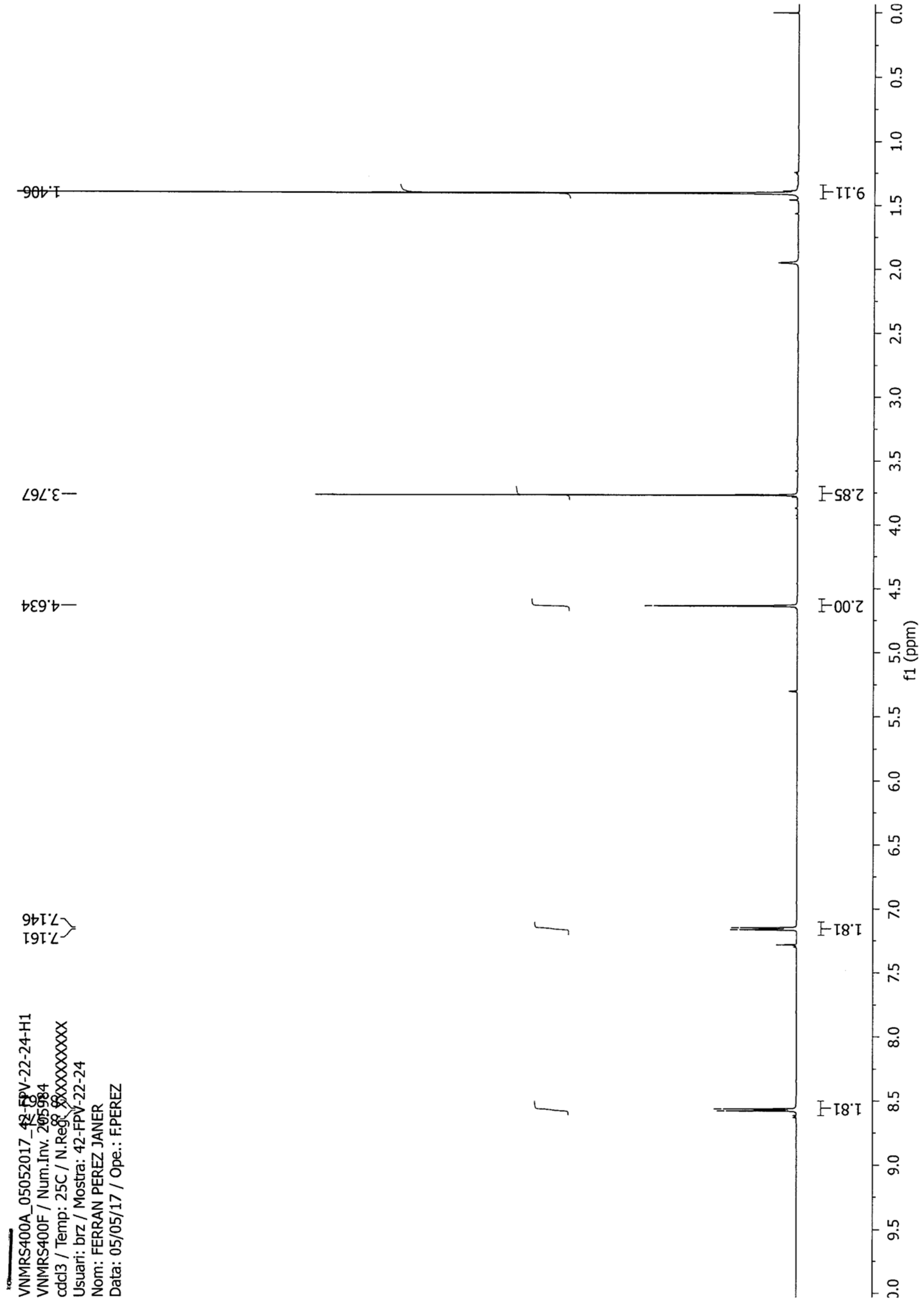
VNMRS400A_05052017_42-FPV-22-24-H1
VNMRS400F / Num.Inv. 205984
cdcl3 / Temp: 25C / N.Reg: XXXXXXXXXX
Usuari: brz / Mostra: 42-FPV-22-24
Nom: FERRAN PEREZ JANER
Data: 05/05/17 / Ope.: F.PEREZ
1.406
3.767
4.634
7.146
7.161
9.11
2.85
2.00
1.81
1.81
f1 (ppm)

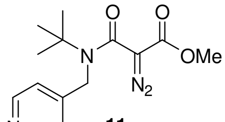

11

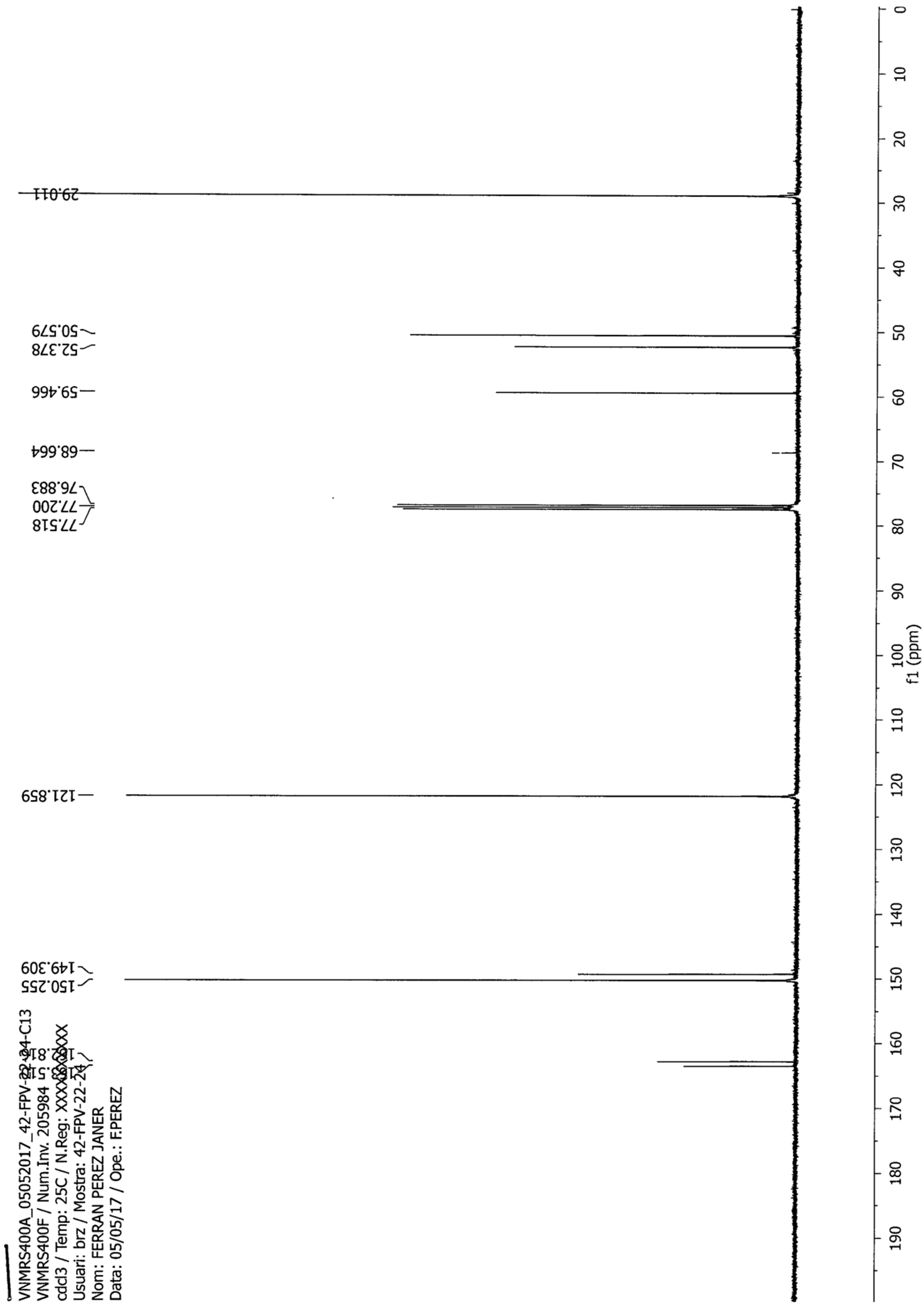
29.011
50.579
52.378
59.466
68.664
76.883
77.200
77.518
121.859
149.309
150.255
VNMRS400A_05052017_42-FPV-22-24-C13
VNMRS400F / Num.Inv. 205984
cdcl3 / Temp: 25C / N.Reg: XXXXXXXX
Usuari: brz / Mostra: 42-FPV-22-24
Nom: FERRAN PEREZ JANER
Data: 05/05/17 / Ope.: F.PEREZ
f1 (ppm)

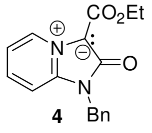

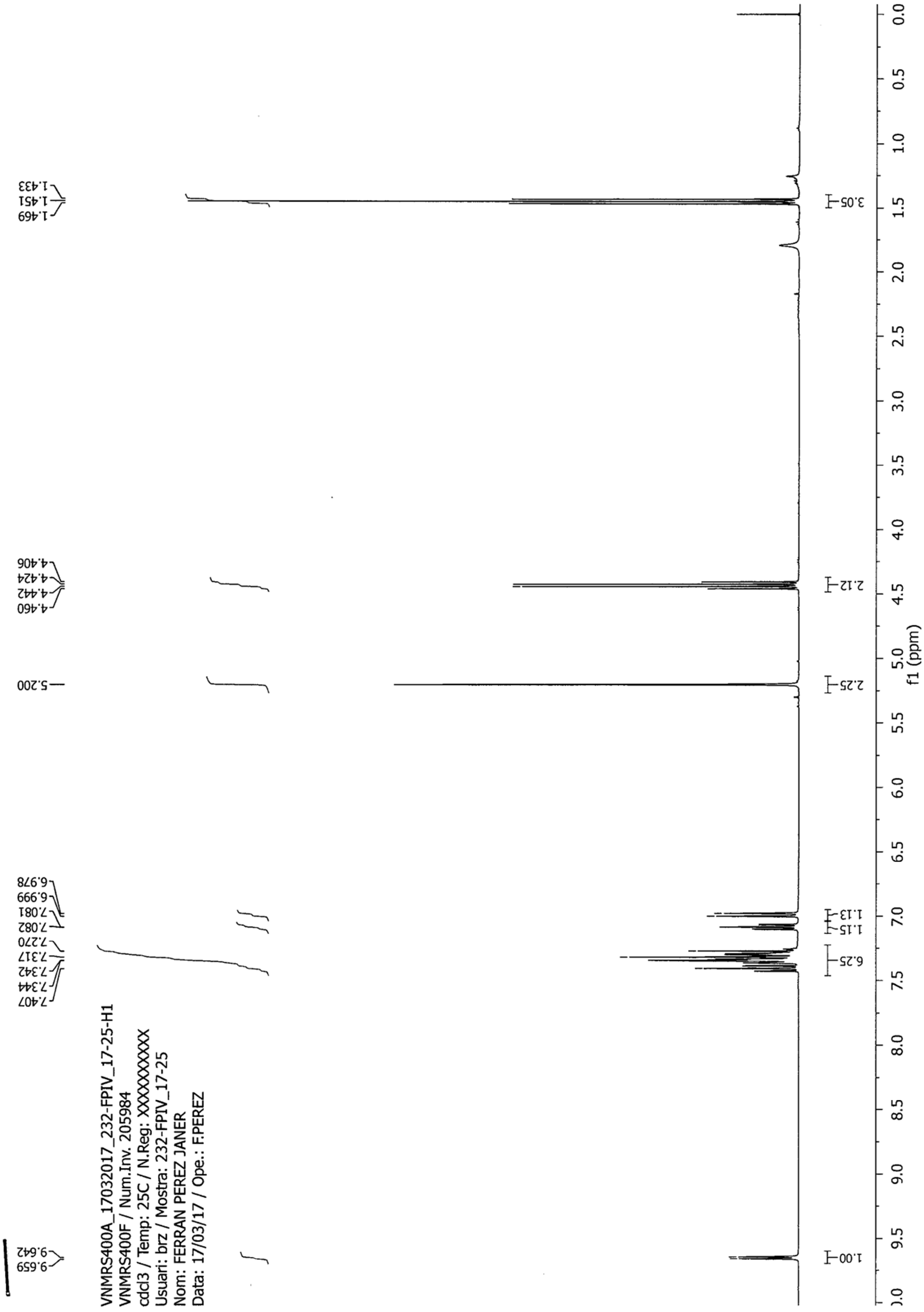
VNMRS400A_17032017_232-FPIV_17-25-H1
VNMRS400F / Num.Inv. 205984
cdcl3 / Temp: 25C / N.Reg: XXXXXXXXXX
Usuari: brz / Mostra: 232-FPIV_17-25
Nom: FERRAN PEREZ JANER
Data: 17/03/17 / Ope.: F.PEREZ
9.659
9.642
7.407
7.344
7.342
7.317
7.270
7.082
7.081
6.999
6.978
5.200
4.460
4.442
4.424
4.406
1.469
1.451
1.433
1.00
6.25
1.15
1.13
2.25
2.12
3.05
f1 (ppm)

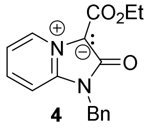
CO2Et
O
N
N
Bn

4

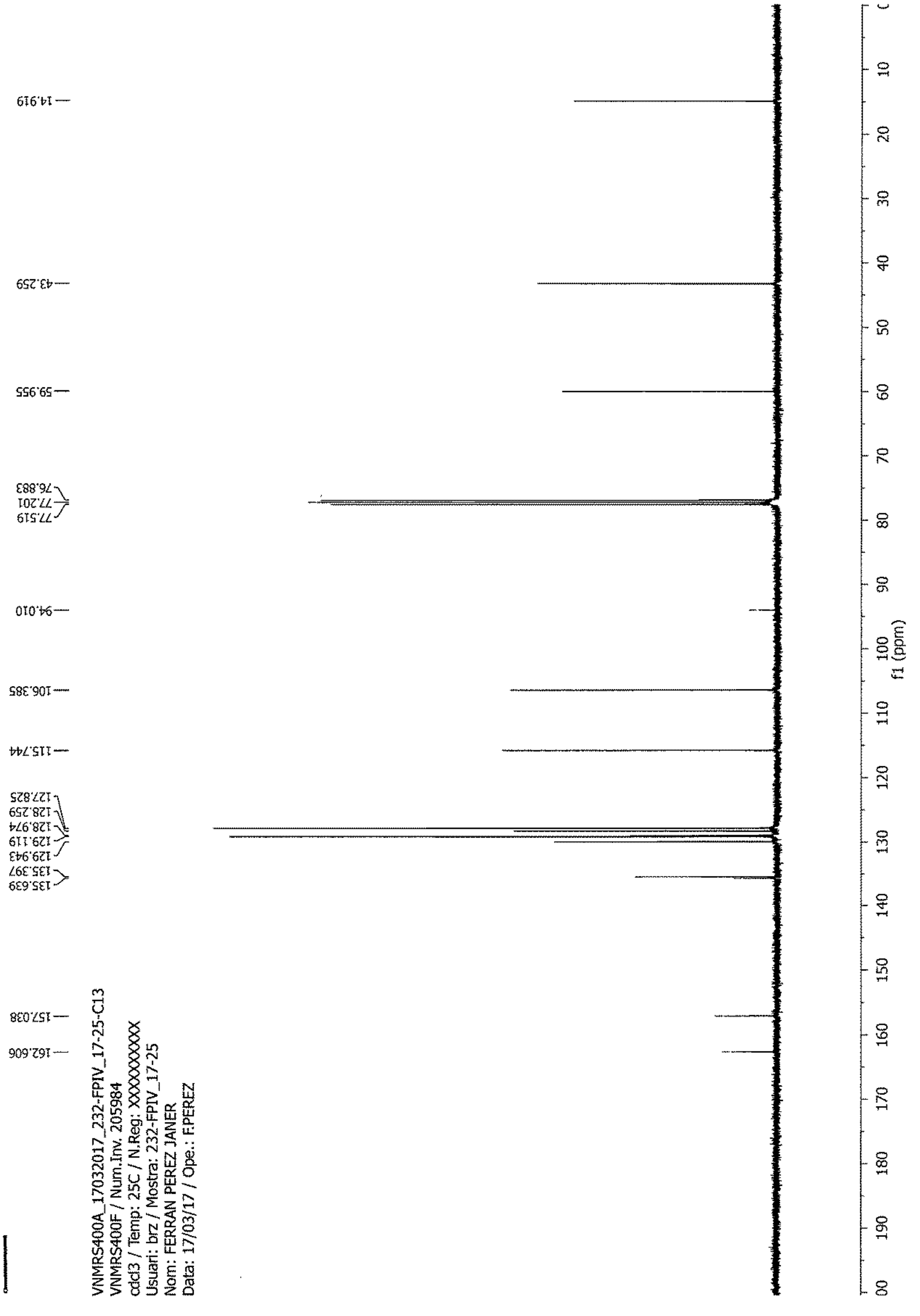
14.919
43.259
59.955
76.883
77.201
77.519
94.010
106.385
115.744
127.825
128.259
128.974
129.119
129.943
135.397
135.639
157.038
162.606
VNMRS400A_17032017_232-FPIV_17-25-C13
VNMRS400F / Num.Inv. 205984
cdcl3 / Temp: 25C / N.Reg: XXXXXXXXXX
Usuari: brz / Mostra: 232-FPIV_17-25
Nom: FERRAN PEREZ JANER
Data: 17/03/17 / Ope.: F.PEREZ
f1 (ppm)

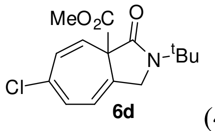

(4.5:1 mixture of ***trans*-7d** and **6d**)

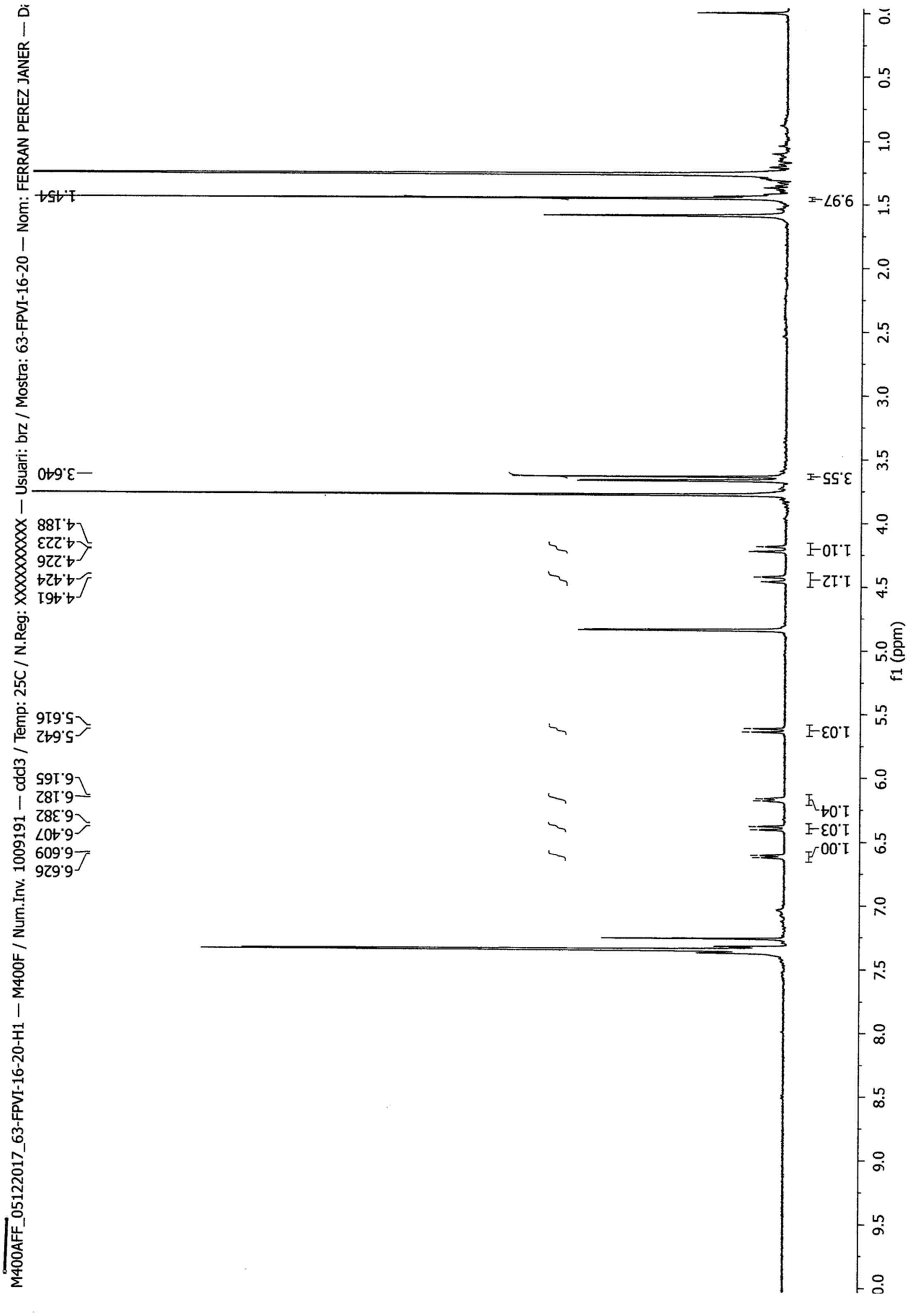

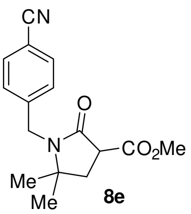
CN
O
N
CO2Me
Me
Me

8e

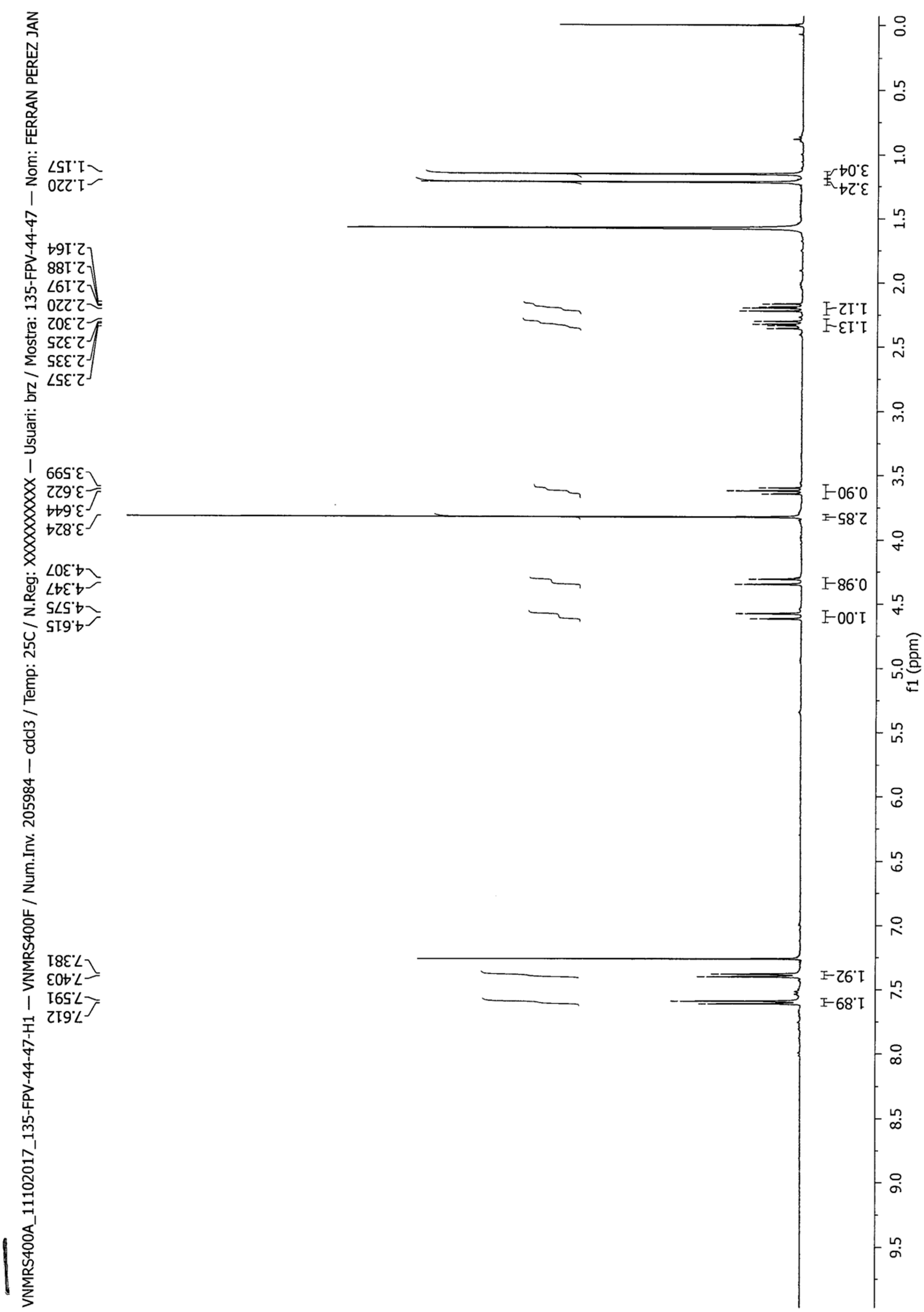
VNMRS400A_11102017_135-FPV-44-47-H1 — VNMRS400F / Num.Inv. 205984 — cdcl3 / Temp: 25C / N.Reg: XXXXXXXXXXX — Usuari: brz / Mostra: 135-FPV-44-47 — Nom: FERRAN PEREZ JAN
1.157
1.220
2.164
2.188
2.197
2.220
2.302
2.325
2.335
2.357
3.599
3.622
3.644
3.824
4.307
4.347
4.575
4.615
7.381
7.403
7.591
7.612
3.04
3.24
1.12
1.13
0.90
2.85
0.98
1.00
1.92
1.89
f1 (ppm)

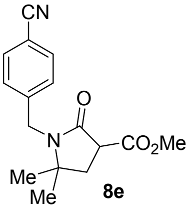
CN
O
N
CO2Me
Me
Me

8e

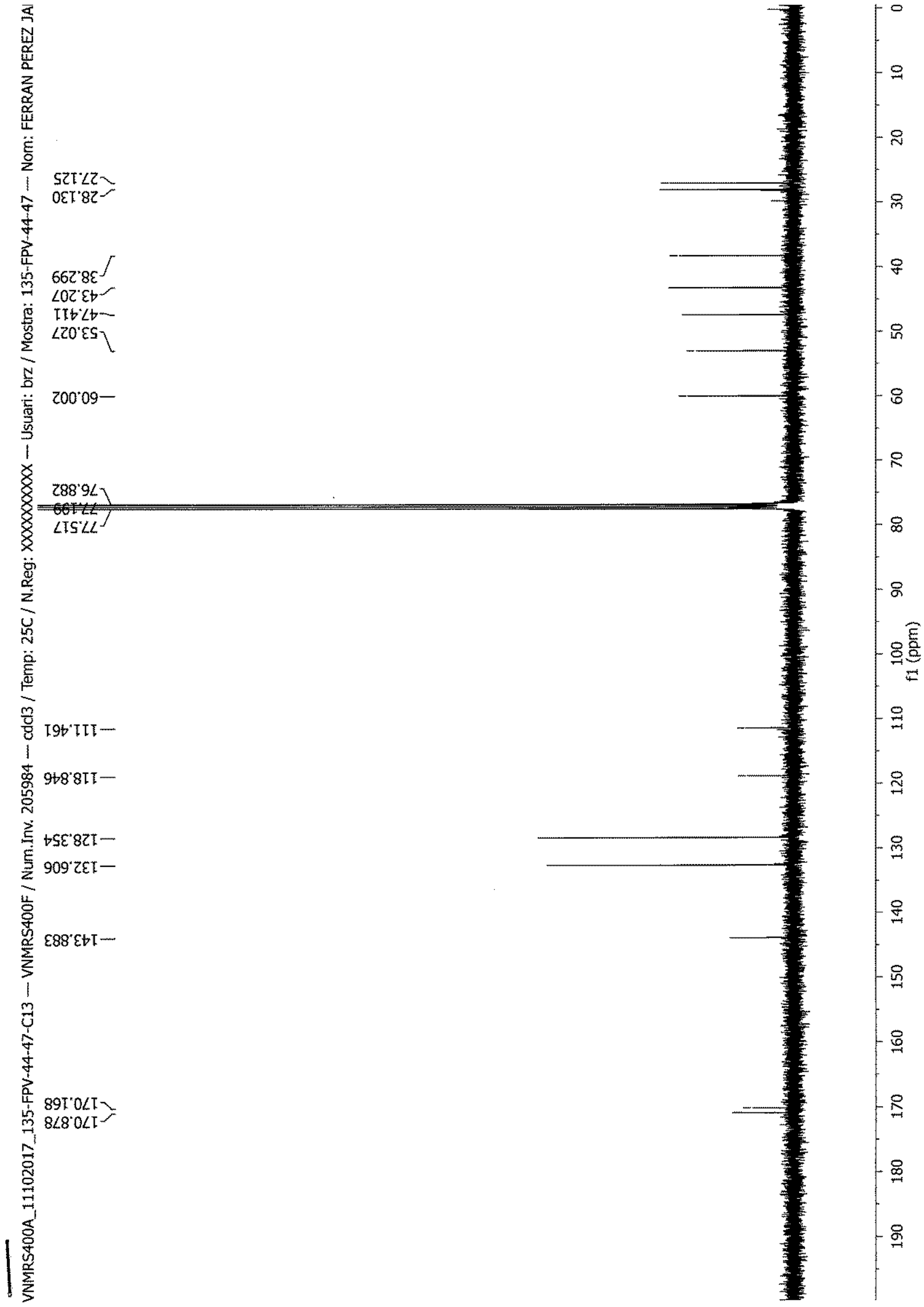
VNMRS400A_11102017_135-FPV-44-47-C13 — VNMRS400F / Num.Inv. 205984 — cdcl3 / Temp: 25C / N.Reg: XXXXXXXXXX — Usuari: brz / Mostra: 135-FPV-44-47 — Nom: FERRAN PEREZ JA
27.125
28.130
38.299
43.207
47.411
53.027
60.002
76.882
77.199
77.517
111.461
118.846
128.354
132.606
143.883
170.168
170.878
f1 (ppm)

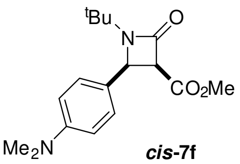
tBu
N
O
CO2Me
Me2N

cis-7f

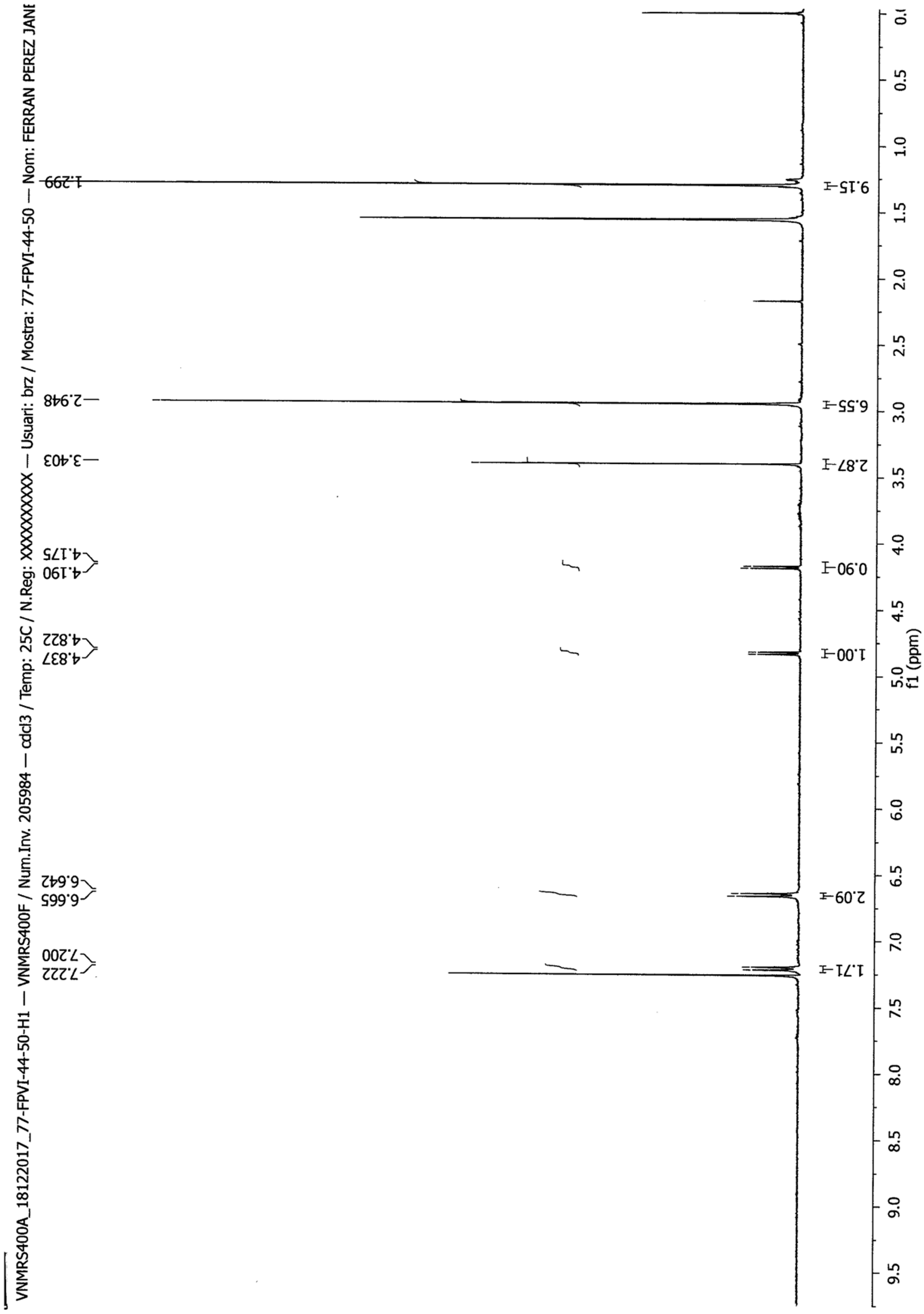
VNMRS400A_18122017_77-FPVI-44-50-H1 — VNMRS400F / Num.Inv. 205984 — cdcl3 / Temp: 25C / N.Reg: XXXXXXXXXX — Usuari: brz / Mostra: 77-FPVI-44-50 — Nom: FERRAN PEREZ JANE
1.299
2.948
3.403
4.175
4.190
4.822
4.837
6.642
6.665
7.200
7.222
9.15
6.55
2.87
0.90
1.00
2.09
1.71
f1 (ppm)

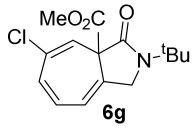

6g

(11:1 mixture of ***trans*-7g** and **6g**)

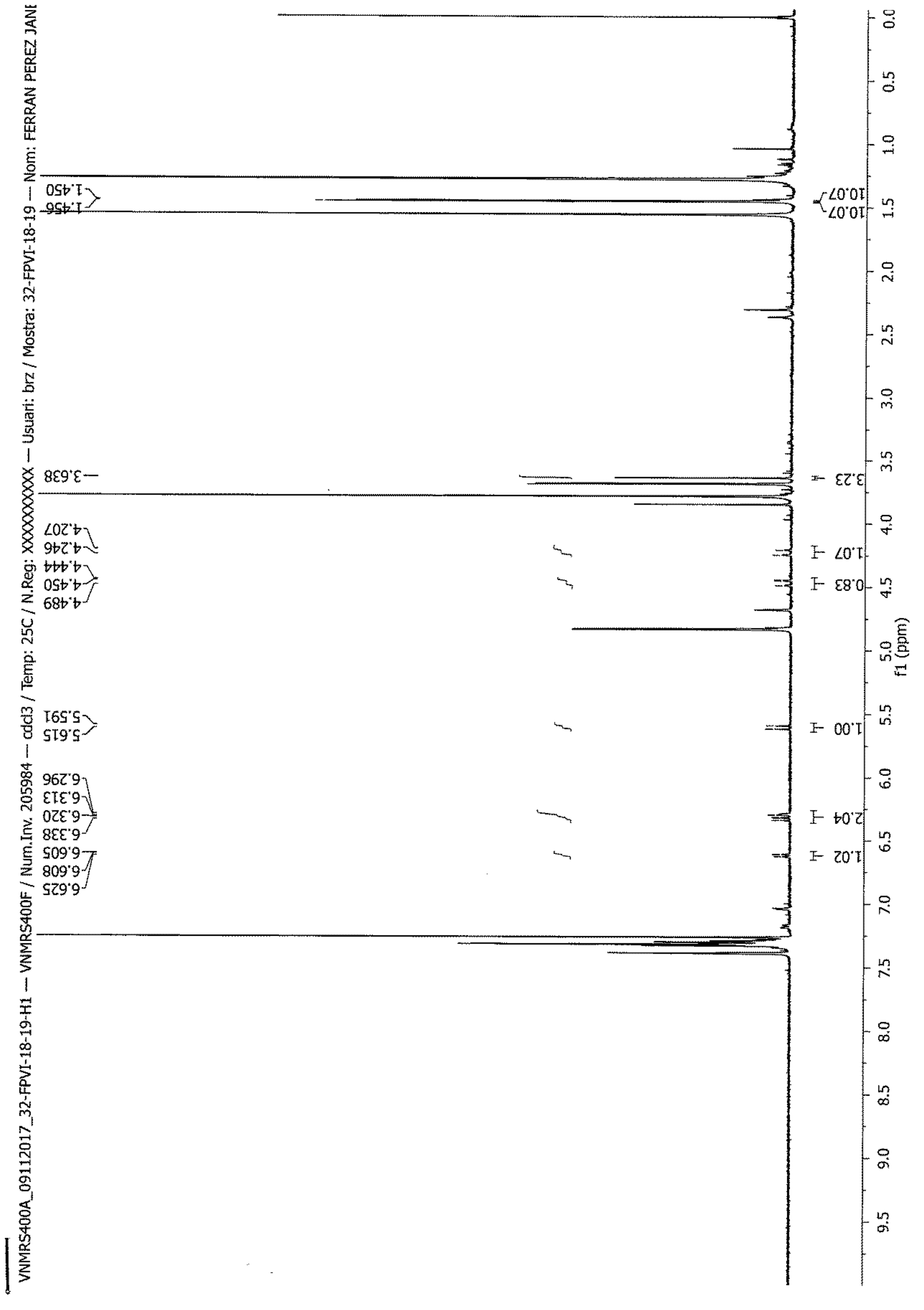

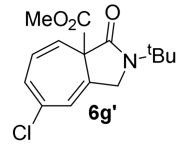

6g'

(12:4:1 mixture of ***trans*-7g, 6g** and **6g'**)

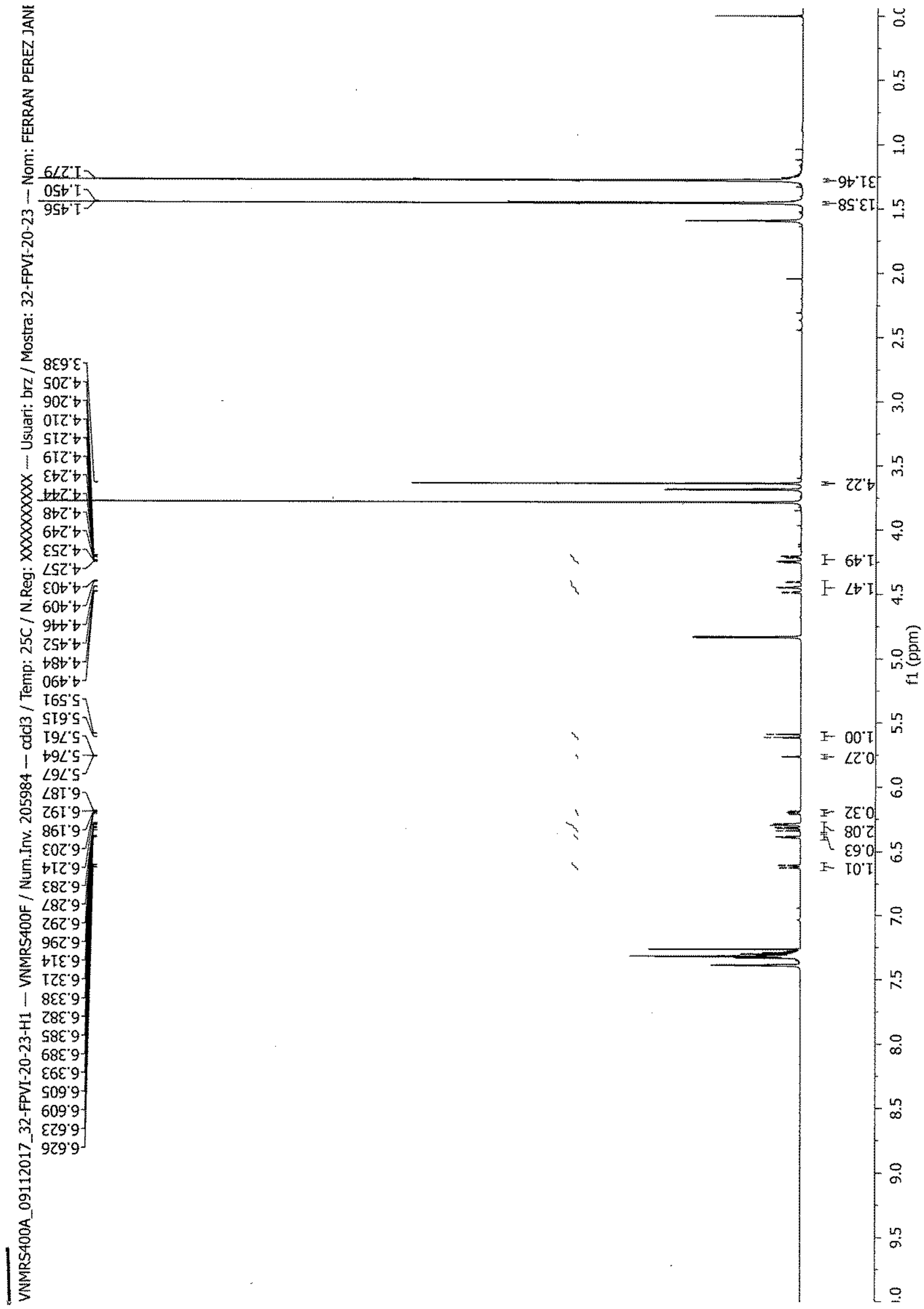

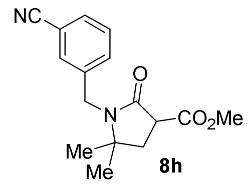

(2:1 mixture of **8h** and *cis*-**7h**)

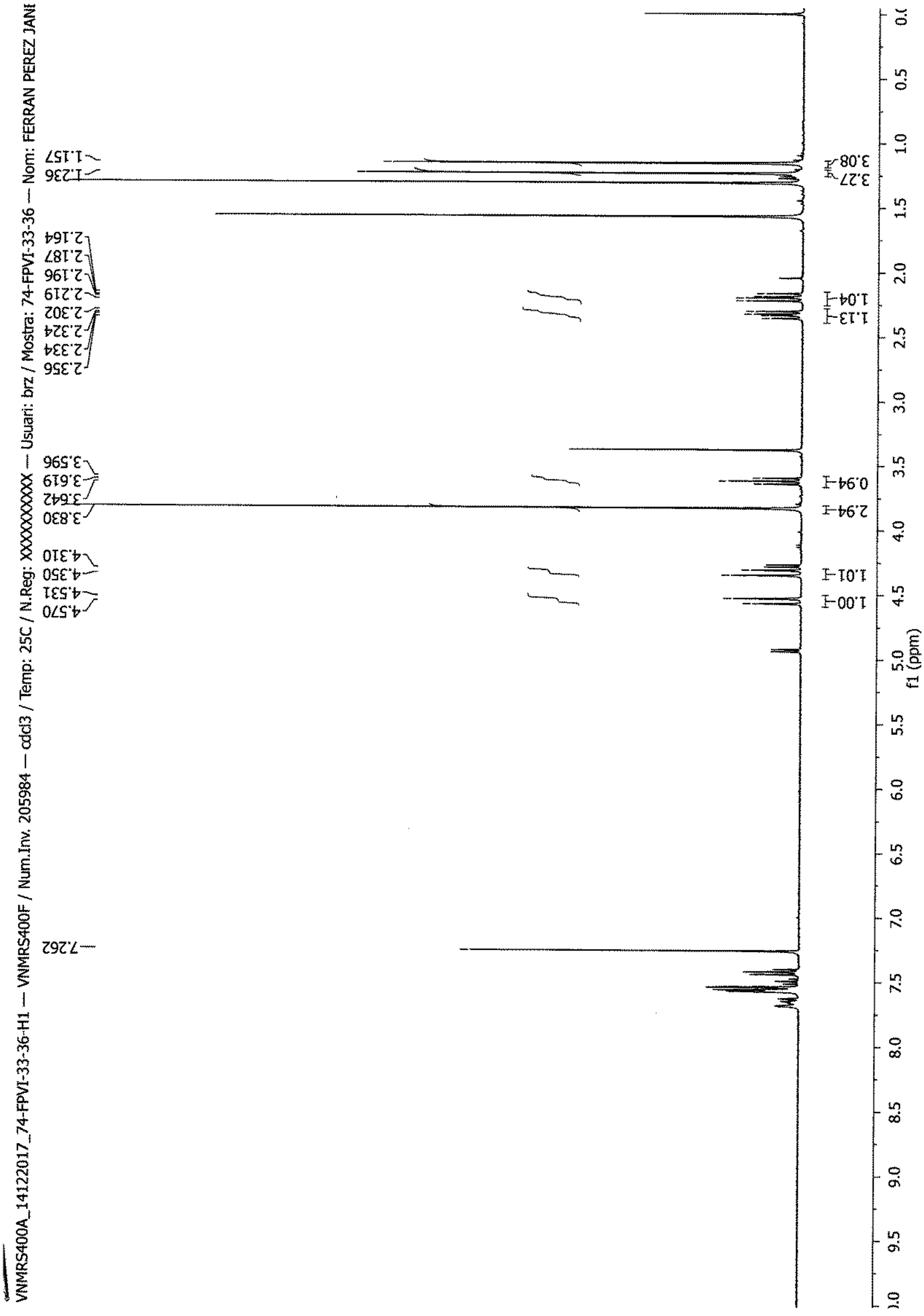

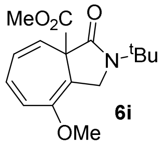

6i

(1.5:1 mixture of **6i** and ***cis*-7i**)

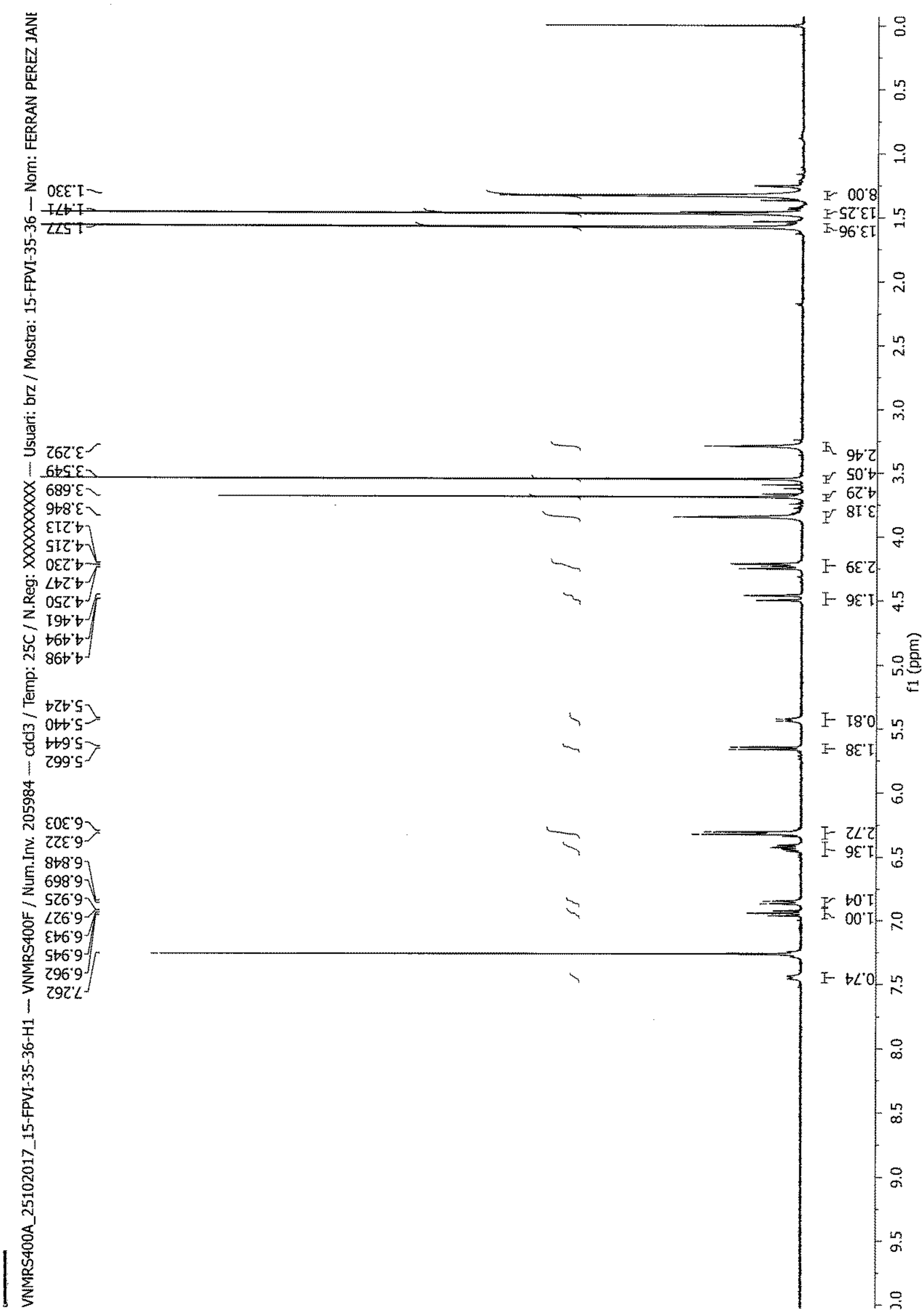

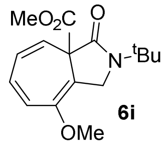

6i (1:8 mixture of **6i** and ***cis*-7i**)

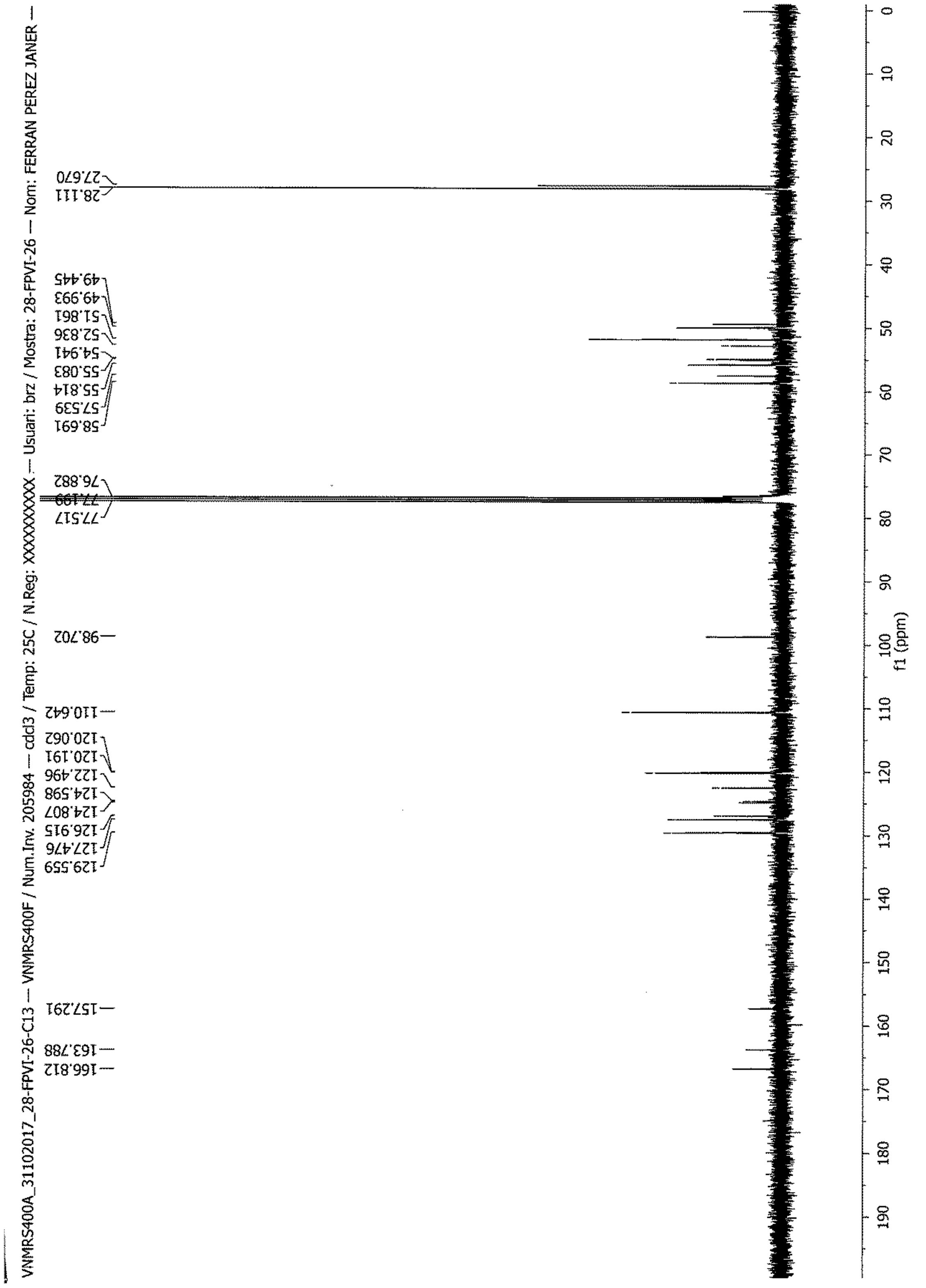

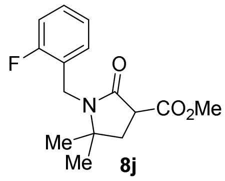

8j

(8:1 mixture of **8j** and ***cis*-7j**)

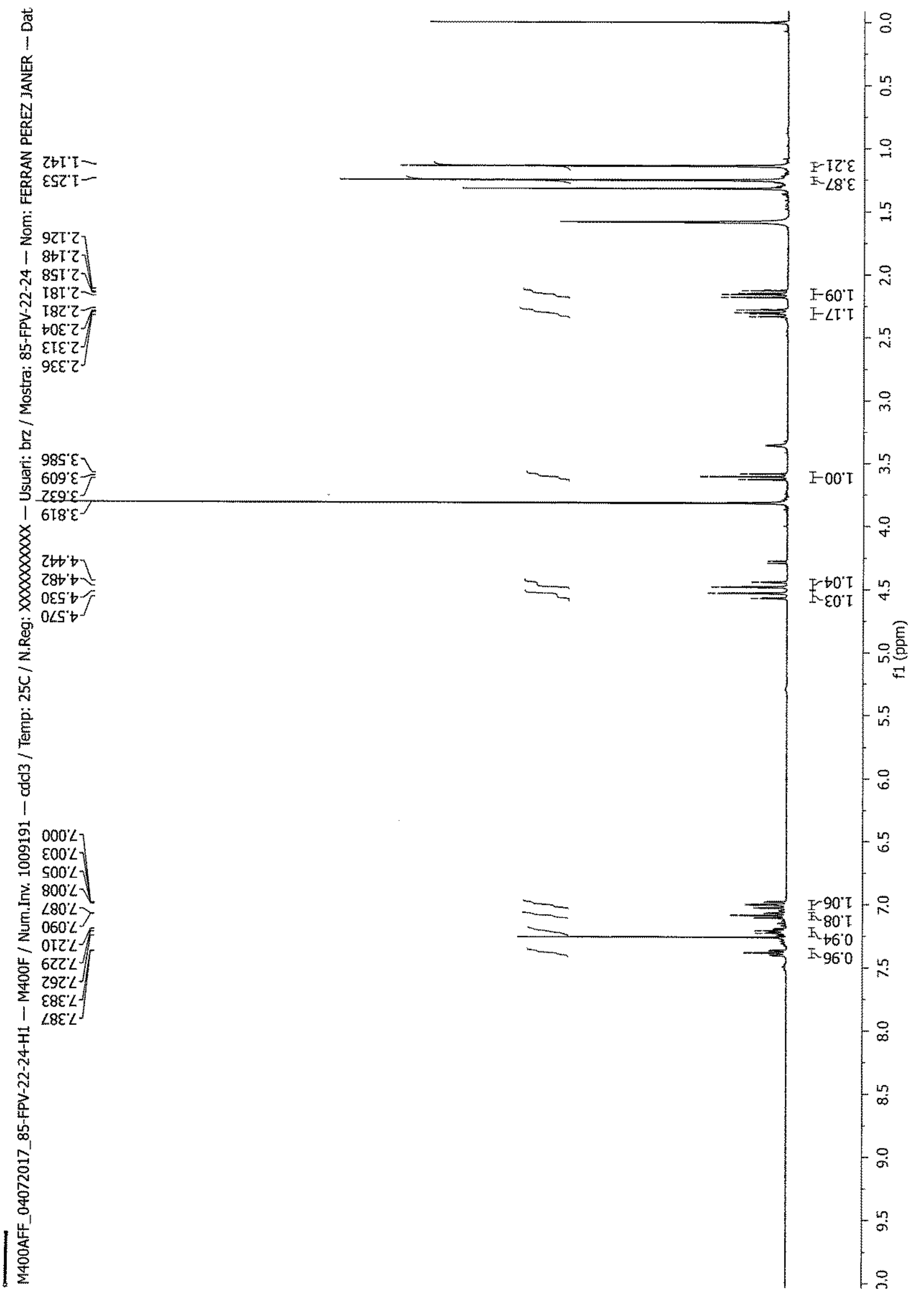

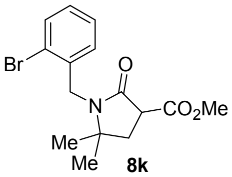
Br
O
N
CO2Me
Me
Me

8k

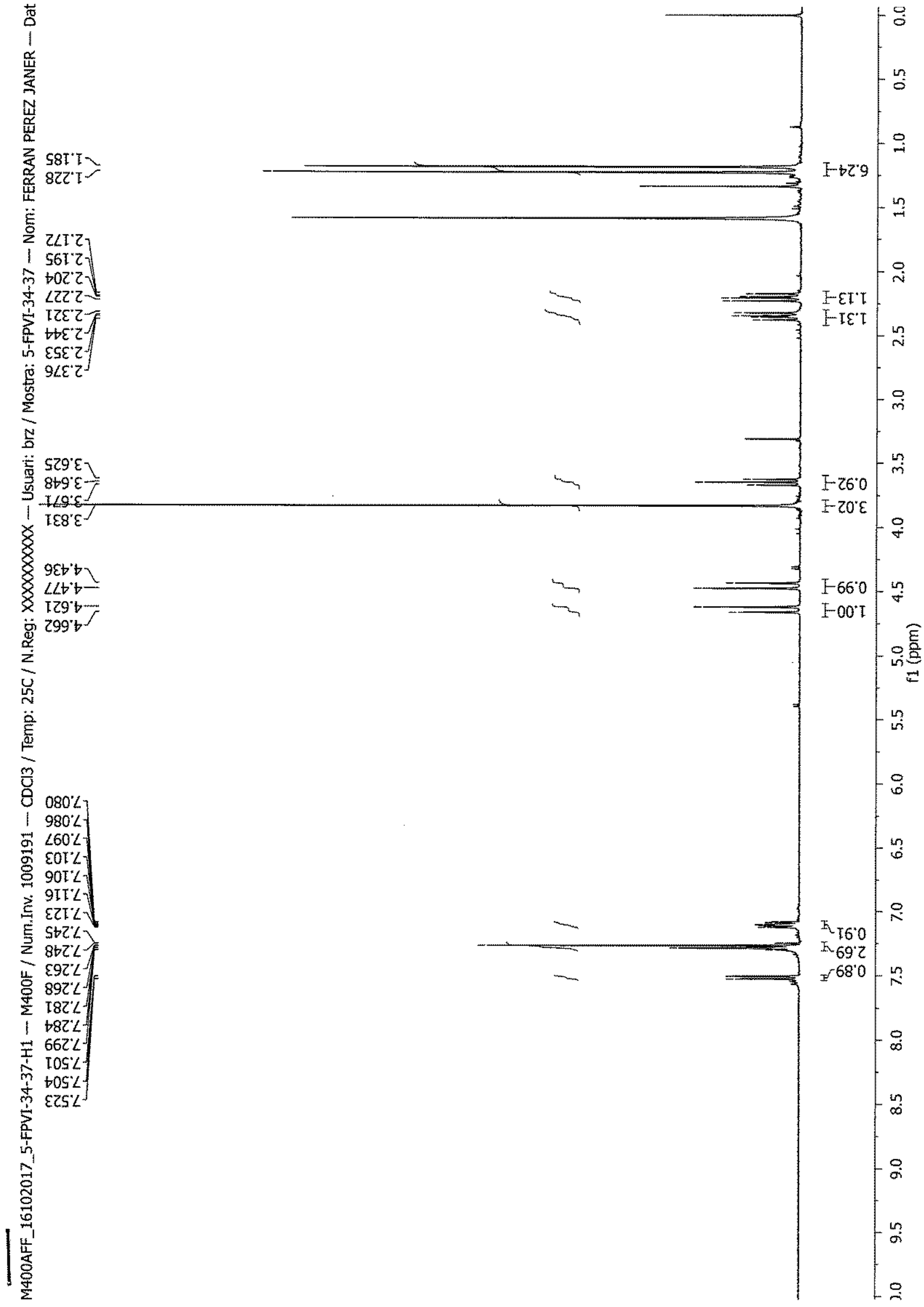
M400AFF_16102017_5-FPVI-34-37-H1 — M400F / Num.Inv. 1009191 — CDCl3 / Temp: 25C / N.Reg: XXXXXXXXXXX — Usuari: brz / Mostra: 5-FPVI-34-37 — Nom: FERRAN PEREZ JANER — Dat
7.523
7.504
7.501
7.299
7.284
7.281
7.268
7.263
7.248
7.245
7.123
7.116
7.106
7.103
7.097
7.086
7.080
4.662
4.621
4.477
4.436
3.831
3.671
3.648
3.625
2.376
2.353
2.344
2.321
2.227
2.204
2.195
2.172
1.228
1.185
0.89
2.69
0.91
1.00
0.99
3.02
0.92
1.31
1.13
6.24
f1 (ppm)

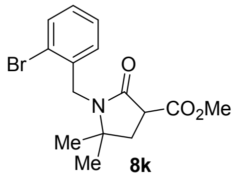

8k

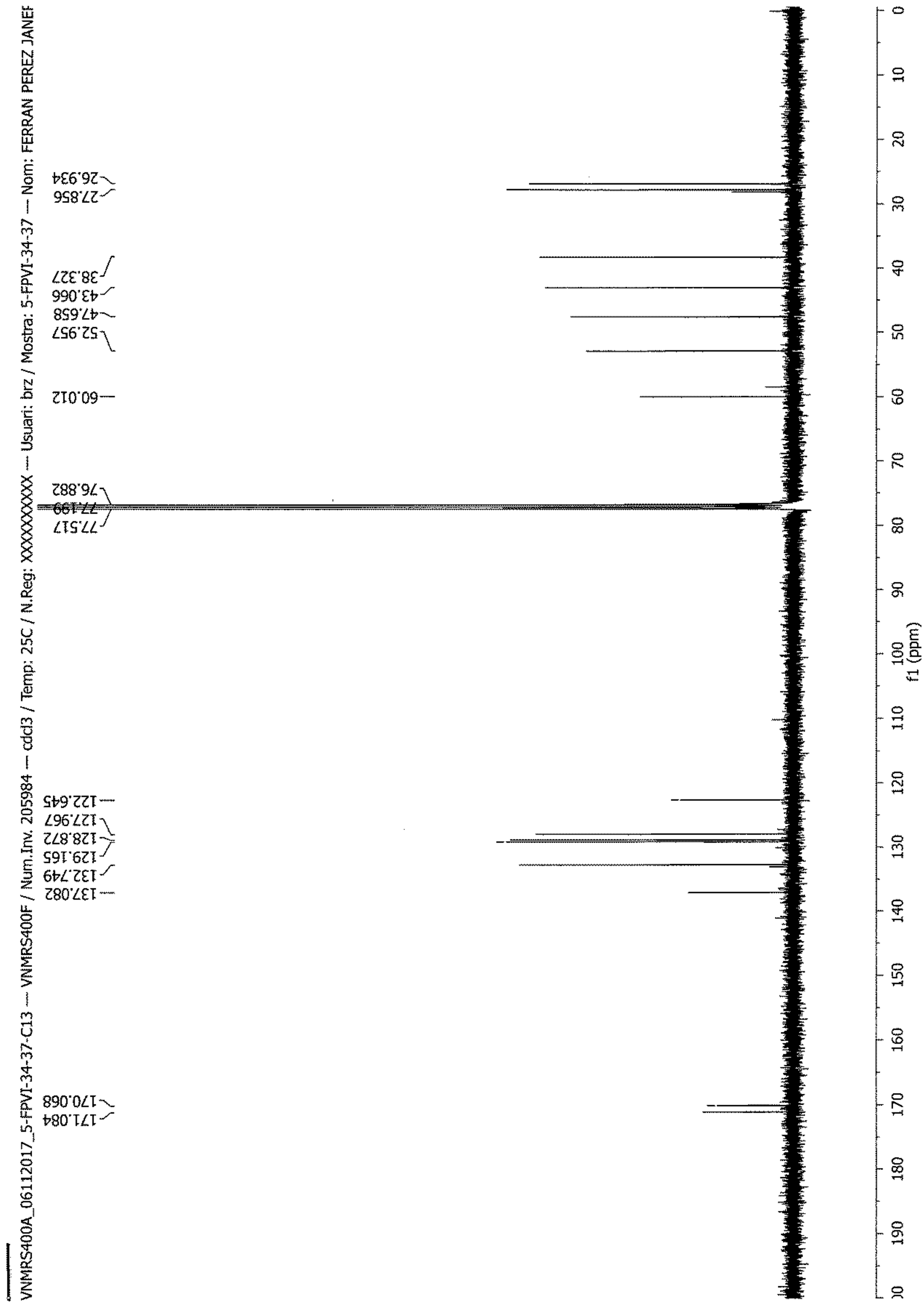

VNMRS400A_06112017_5-FPVI-34-37-C13 — VNMRS400F / Num.Inv. 205984 — cdcl3 / Temp: 25C / N.Reg: XXXXXXXXXX — Usuari: brz / Mostra: 5-FPVI-34-37 — Nom: FERRAN PEREZ JANEF
26.934
27.856
38.327
43.066
47.658
52.957
60.012
76.882
77.199
77.517
122.645
127.967
128.872
129.165
132.749
137.082
170.068
171.084
f1 (ppm)

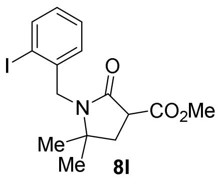

I
O
N
CO_2Me
Me
Me

8l

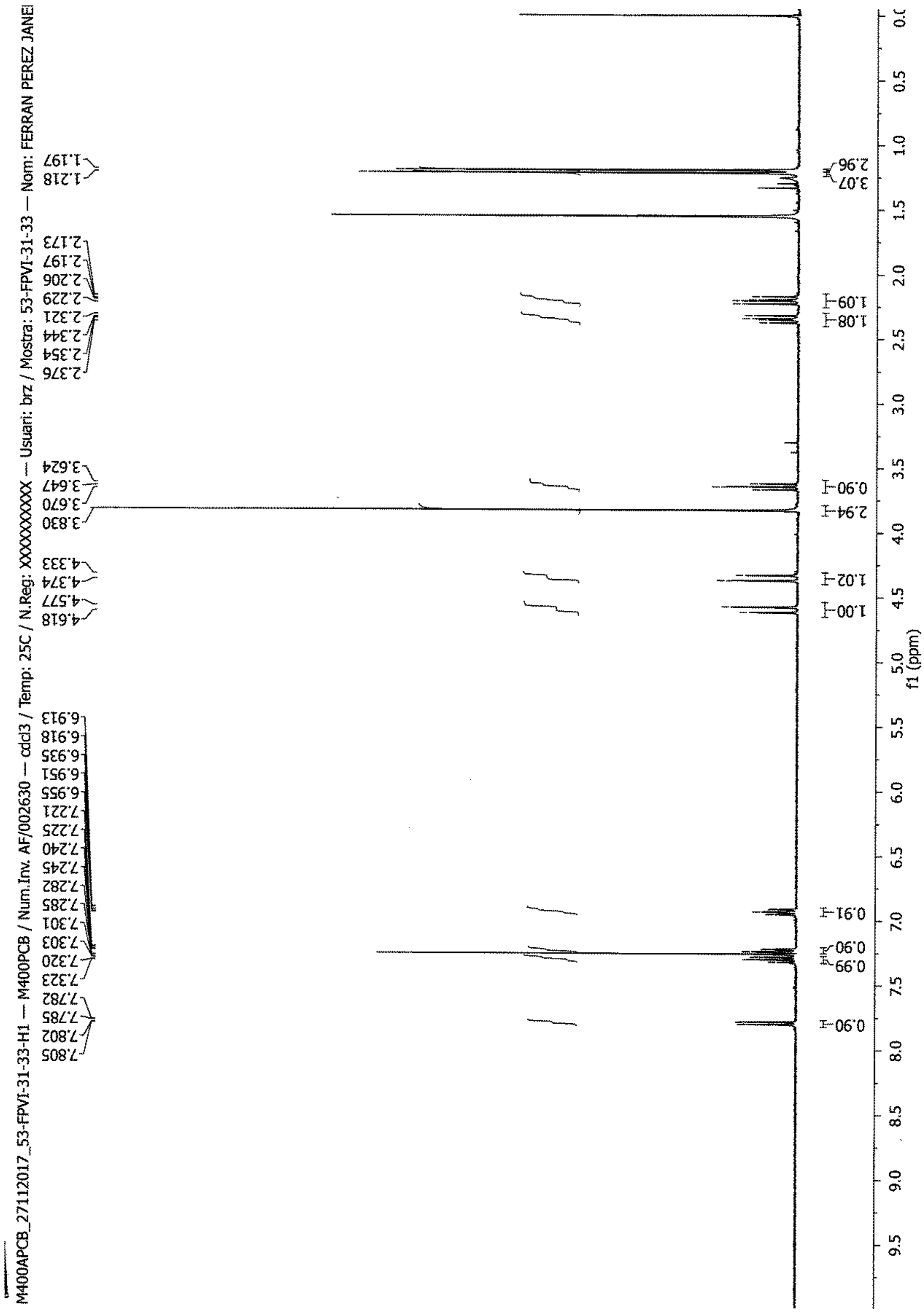

M400APCB_27112017_53-FPVI-31-33-H1 — M400PCB / Num.Inv. AF/002630 — cdcl3 / Temp: 25C / N.Reg: XXXXXXXXXX — Usuari: brz / Mostra: 53-FPVI-31-33 — Nom: FERRAN PEREZ JANEI
1.197
1.218
2.173
2.197
2.206
2.229
2.321
2.344
2.354
2.376
3.624
3.647
3.670
3.830
4.333
4.374
4.577
4.618
6.913
6.918
6.935
6.951
6.955
7.221
7.225
7.240
7.245
7.282
7.285
7.301
7.303
7.320
7.323
7.782
7.785
7.802
7.805
2.96
3.07
1.09
1.08
0.90
2.94
1.02
1.00
0.91
0.90
0.99
0.90
0.0
0.5
1.0
1.5
2.0
2.5
3.0
3.5
4.0
4.5
5.0
f1 (ppm)
5.5
6.0
6.5
7.0
7.5
8.0
8.5
9.0
9.5

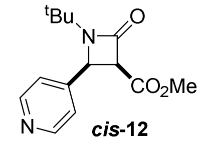

cis-**12**

(2.6:1 mixture of ***trans*-12** and ***cis*-12**)

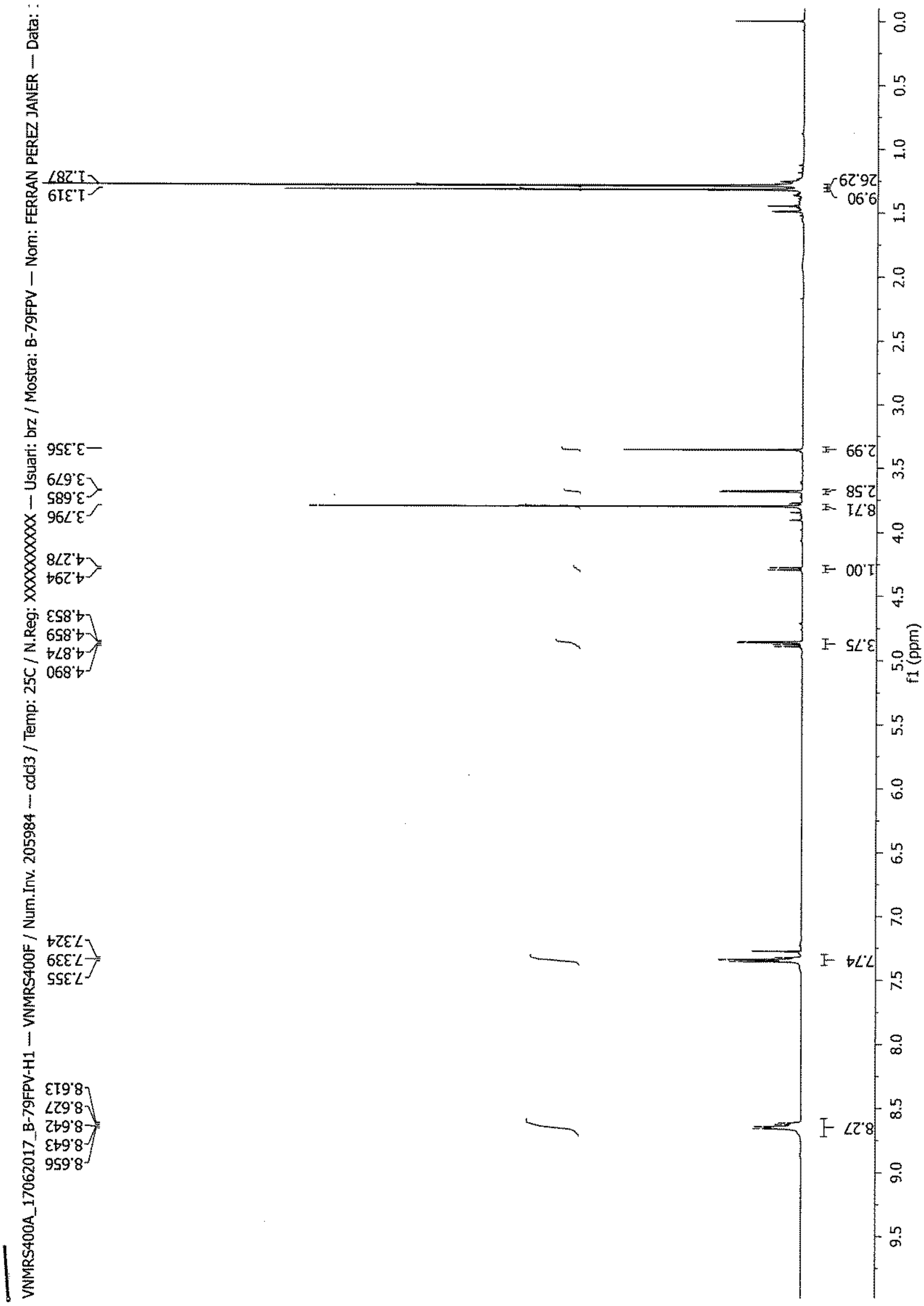

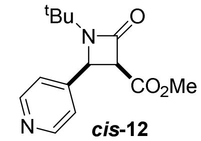

***cis*-12**

(2.6:1 mixture of ***trans*-12** and ***cis*-12**)

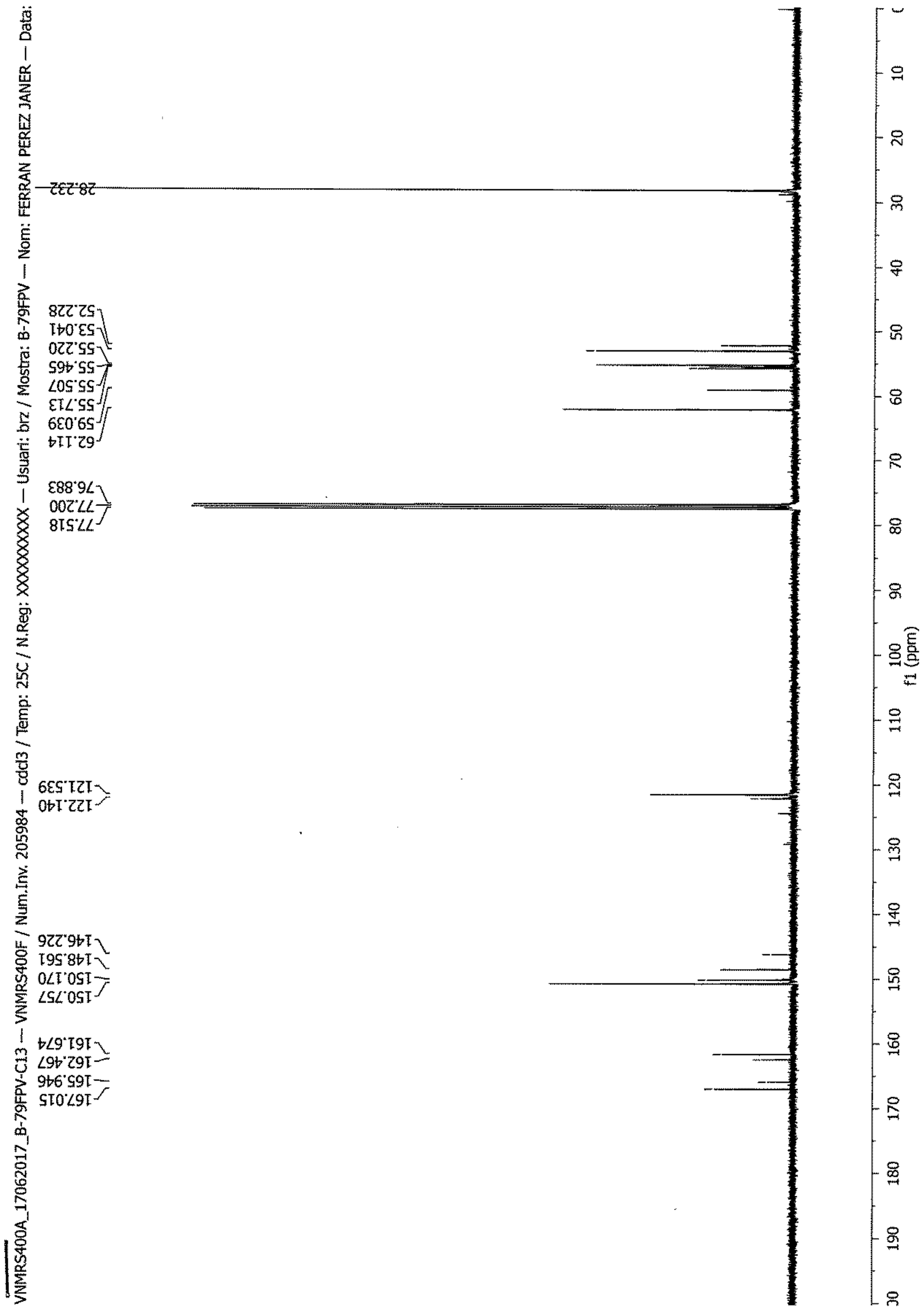

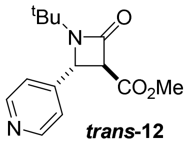

^{t}Bu
N
O
CO_2Me
N

***trans*-12**

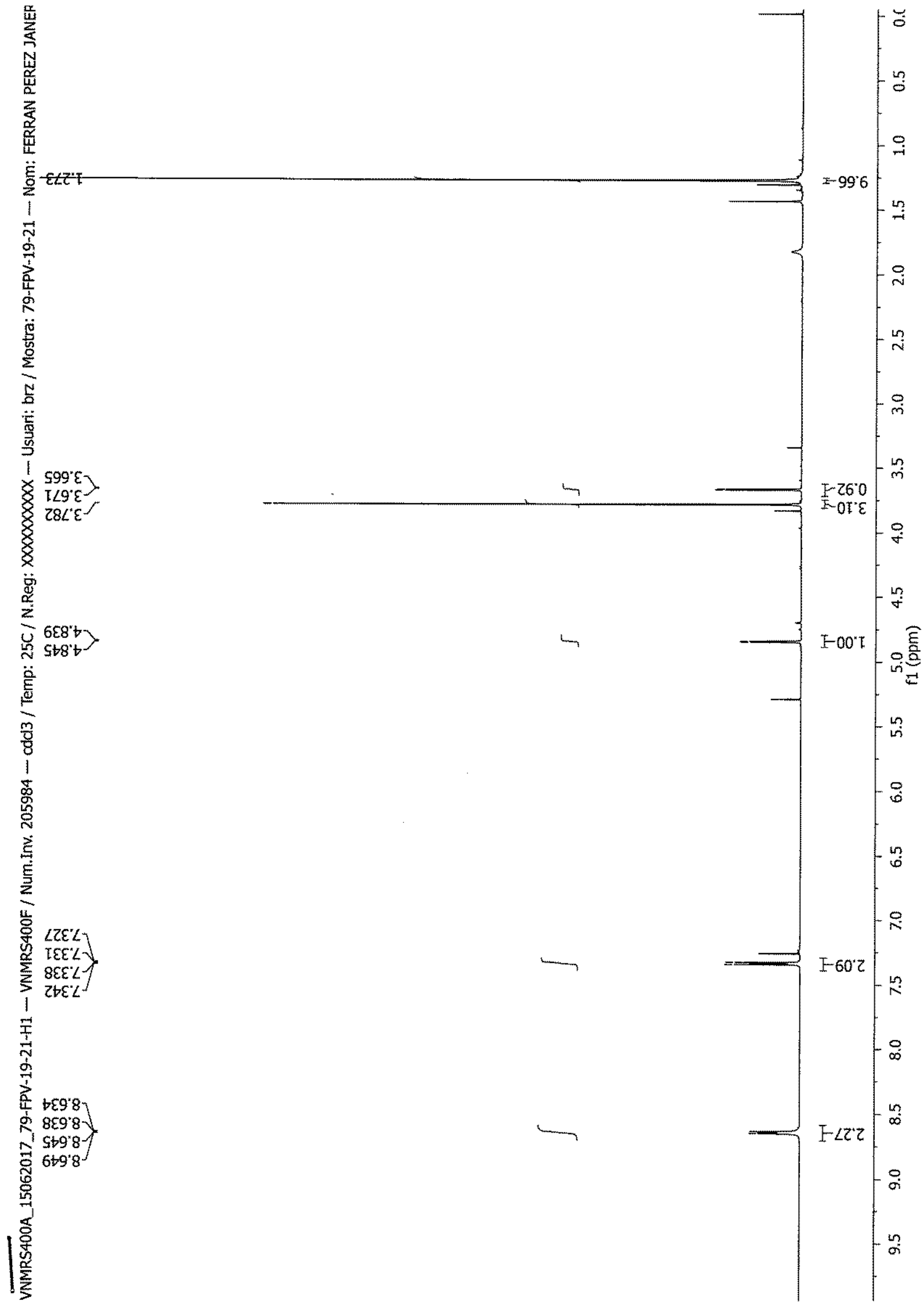

VNMRS400A_15062017_79-FPV-19-21-H1 — VNMRS400F / Num.Inv. 205984 — cdcl3 / Temp: 25C / N.Reg: XXXXXXXXXX — Usuari: brz / Mostra: 79-FPV-19-21 — Nom: FERRAN PEREZ JANER
1.273
3.665
3.671
3.782
4.839
4.845
7.327
7.331
7.338
7.342
8.634
8.638
8.645
8.649
9.66
0.92
3.10
1.00
2.09
2.27
f1 (ppm)

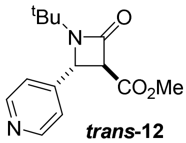

tBu
N
O
CO2Me
N

***trans*-12**

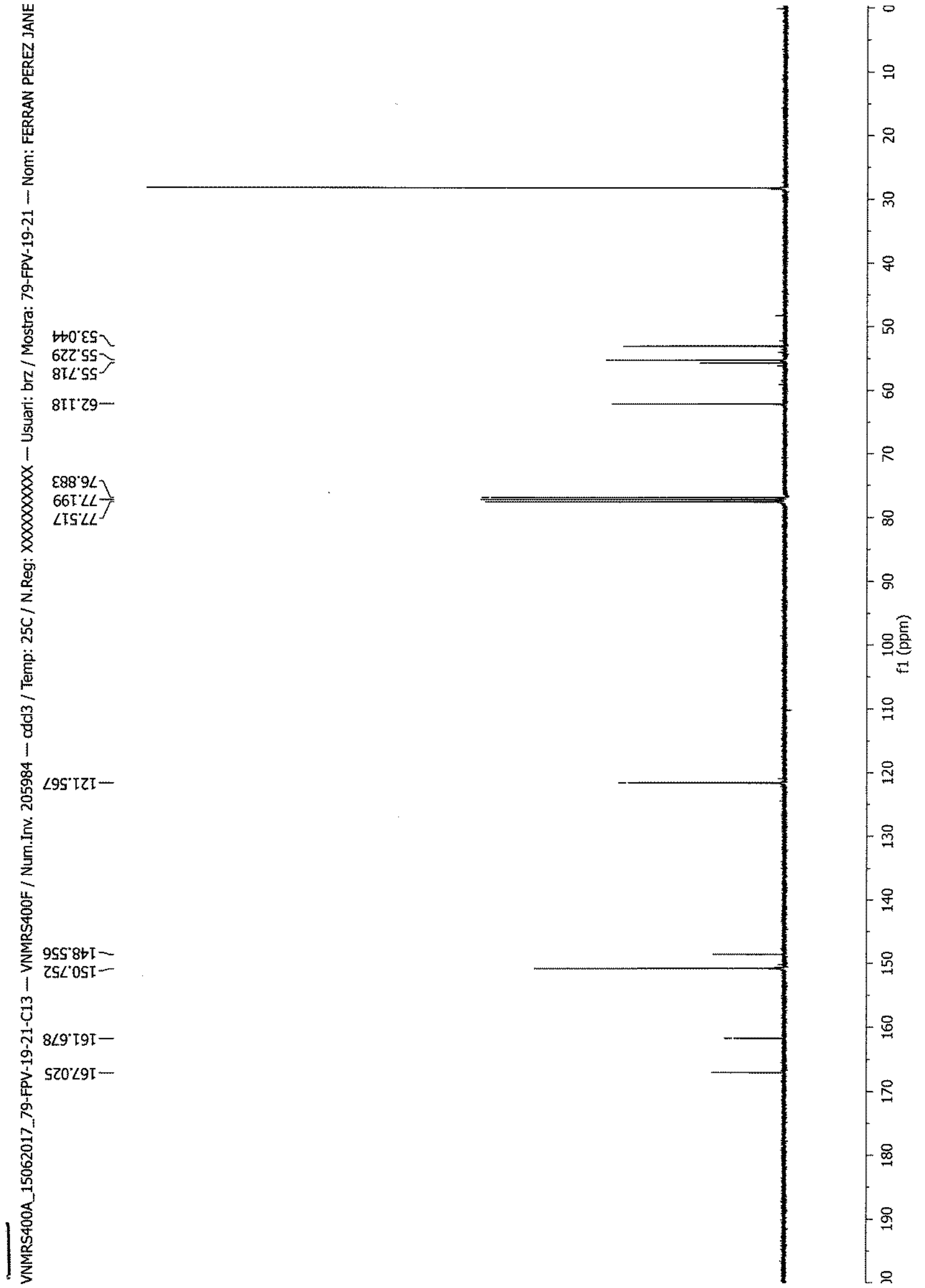

VNMRS400A_15062017_79-FPV-19-21-C13 — VNMRS400F / Num.Inv. 205984 — cdcl3 / Temp: 25C / N.Reg: XXXXXXXXXX — Usuari: brz / Mostra: 79-FPV-19-21 — Nom: FERRAN PEREZ JANE
53.044
55.229
55.718
62.118
76.883
77.199
77.517
121.567
148.556
150.752
161.678
167.025
f1 (ppm)

Cartesian coordinates and total energies

Cartesian coordinates (in Å) and total energies (in a.u.) of all the stationary points discussed in the text. All calculations have been performed at the PCM(solvent)-B3LYP-D3/def2-TZVPP//PCM(solvent)-B3LYP-D3/def2-SVP.

INT0: E= -1523.093134

Pd	-0.241439000	0.345772000	0.083159000
C	1.433306000	-0.236019000	-2.085325000
C	0.936007000	1.038272000	-1.712257000
C	1.831880000	2.015780000	-1.160842000
C	3.185230000	1.660819000	-0.994192000
C	3.636236000	0.389341000	-1.352974000
C	2.771455000	-0.570264000	-1.893132000
H	0.754809000	-0.941594000	-2.568395000
H	3.141570000	-1.559485000	-2.163226000
H	4.687270000	0.136601000	-1.193816000
H	3.886844000	2.377486000	-0.567218000
N	1.367216000	3.289659000	-0.854835000
C	2.276853000	4.422508000	-0.951834000
H	1.674106000	5.335610000	-1.024740000
H	2.930308000	4.519438000	-0.065131000
H	2.907802000	4.324546000	-1.846357000
C	0.157350000	3.498512000	-0.103471000
O	-0.191855000	4.661814000	0.128857000
C	-0.480452000	2.321339000	0.379443000
C	-1.574418000	2.137820000	1.213277000
O	-1.861736000	0.901822000	1.460218000
O	-2.317554000	3.111047000	1.760623000
C	-3.376204000	2.709439000	2.631612000
H	-3.844135000	3.635460000	2.989735000
H	-4.120715000	2.096546000	2.099736000
H	-2.993046000	2.130269000	3.486689000
H	-0.002659000	1.394512000	-2.147457000
C	-0.294431000	-1.782907000	0.099315000
C	-1.142167000	-3.900908000	0.096645000
C	0.156340000	-3.986375000	0.481112000
H	-1.899571000	-4.667011000	-0.040851000
H	0.757238000	-4.842420000	0.772731000
C	-2.661697000	-2.036167000	-0.561232000
C	-2.739208000	-1.231437000	-1.703611000
C	-3.810881000	-2.339593000	0.176194000
C	-3.975201000	-0.713042000	-2.097135000
H	-1.834715000	-1.017419000	-2.274111000
C	-5.045861000	-1.827779000	-0.232122000
H	-3.731568000	-2.957921000	1.072632000
C	-5.129609000	-1.009638000	-1.363710000
H	-4.035681000	-0.079494000	-2.985049000
H	-5.943370000	-2.059670000	0.345992000
H	-6.094876000	-0.603574000	-1.675112000
C	2.003547000	-2.351351000	0.828881000
C	2.264567000	-1.246923000	1.647732000
C	3.056038000	-3.134857000	0.339827000
C	3.586087000	-0.907792000	1.945172000

H	1.435625000	-0.657878000	2.042291000
C	4.373879000	-2.801776000	0.662295000
H	2.846171000	-3.984891000	-0.312308000
C	4.643369000	-1.682166000	1.456139000
H	3.787061000	-0.035287000	2.570950000
H	5.193325000	-3.410946000	0.273791000
H	5.675376000	-1.414609000	1.694720000
N	-1.395956000	-2.551532000	-0.136893000
N	0.658185000	-2.685305000	0.479833000

TS1: E= -1523.068328

Pd	-0.186123000	0.846867000	-0.406410000
C	0.633259000	3.698845000	0.259751000
C	1.085568000	2.371001000	0.352269000
C	2.470014000	2.060890000	0.217754000
C	3.350881000	3.111910000	-0.126949000
C	2.868475000	4.413891000	-0.274567000
C	1.516328000	4.727253000	-0.072228000
H	-0.426845000	3.912504000	0.425759000
H	1.163218000	5.755992000	-0.174748000
H	3.573840000	5.207822000	-0.535397000
H	4.416347000	2.928017000	-0.259258000
N	2.919041000	0.765506000	0.453465000
C	4.320091000	0.449421000	0.225524000
H	4.986890000	0.985585000	0.923634000
H	4.452331000	-0.625624000	0.377209000
H	4.607157000	0.708281000	-0.805117000
C	2.136662000	-0.223571000	1.148143000
O	2.732482000	-1.183101000	1.641418000
C	0.713071000	0.006413000	1.239670000
C	-0.225487000	-0.682630000	2.096164000
O	-1.442730000	-0.500502000	2.097332000
O	0.333373000	-1.568332000	2.966088000
C	-0.570550000	-2.232183000	3.840767000
H	0.038425000	-2.909350000	4.454746000
H	-1.105568000	-1.519064000	4.488882000
H	-1.321915000	-2.810331000	3.278697000
H	0.631807000	1.477162000	1.211064000
C	-1.193759000	-0.874694000	-0.933693000
C	-2.793307000	-2.490219000	-0.952375000
C	-1.594336000	-3.104374000	-1.126181000
H	-3.802422000	-2.890375000	-0.925402000
H	-1.344008000	-4.148938000	-1.285181000
N	-0.629197000	-2.101100000	-1.125175000
N	-2.529620000	-1.127806000	-0.853015000
C	-3.517465000	-0.129622000	-0.601029000
C	-3.537475000	1.038092000	-1.372972000
C	-4.451861000	-0.324103000	0.421424000
C	-4.494909000	2.021381000	-1.109964000
H	-2.811396000	1.163737000	-2.178559000
C	-5.415319000	0.657344000	0.667105000
H	-4.398707000	-1.222978000	1.037456000
C	-5.437403000	1.831947000	-0.093242000
H	-4.510049000	2.933641000	-1.710969000
H	-6.142273000	0.509164000	1.469100000
H	-6.188124000	2.599889000	0.107437000
C	0.774843000	-2.340225000	-1.261511000
C	1.384585000	-3.326447000	-0.481828000
C	1.525430000	-1.584061000	-2.169451000
C	2.755551000	-3.557518000	-0.614012000

H	0.794763000	-3.880695000	0.250362000
C	2.898202000	-1.810081000	-2.281914000
H	1.031072000	-0.823831000	-2.775821000
C	3.515270000	-2.799759000	-1.508936000
H	3.235076000	-4.316128000	0.008337000
H	3.486965000	-1.212060000	-2.981420000
H	4.590369000	-2.972425000	-1.597893000

TS1′: E= −1523.050332

Pd	-0.356750000	-0.882021000	-0.421662000
C	0.551743000	-0.018129000	1.210155000
N	2.520131000	-1.063990000	0.383357000
C	1.996328000	0.093328000	1.047417000
O	2.708427000	1.000400000	1.459839000
C	1.500855000	-2.040741000	0.061785000
H	1.406815000	-2.241052000	-1.023122000
H	0.604559000	-1.467144000	0.967004000
C	-0.210164000	0.781965000	2.129492000
O	0.208177000	1.658974000	2.874302000
O	-1.550848000	0.461034000	2.135269000
C	-2.384872000	1.249684000	2.972385000
H	-2.085198000	1.176325000	4.029835000
H	-2.357162000	2.311386000	2.678281000
H	-3.403152000	0.858593000	2.843214000
C	-1.786110000	0.551405000	-0.718001000
C	-3.720107000	1.740063000	-0.695560000
C	-2.699400000	2.626886000	-0.832300000
H	-4.793744000	1.889130000	-0.629726000
H	-2.697919000	3.708398000	-0.930414000
N	-3.142559000	0.474922000	-0.630780000
N	-1.526681000	1.880239000	-0.856509000
C	-3.886046000	-0.734635000	-0.441268000
C	-3.571991000	-1.582578000	0.628026000
C	-4.928284000	-1.044143000	-1.322275000
C	-4.302463000	-2.762232000	0.798327000
H	-2.773790000	-1.297365000	1.314077000
C	-5.659671000	-2.220640000	-1.134159000
H	-5.154623000	-0.372896000	-2.153447000
C	-5.344766000	-3.083490000	-0.078635000
H	-4.061380000	-3.427629000	1.630807000
H	-6.471932000	-2.466728000	-1.822070000
H	-5.914650000	-4.004988000	0.062268000
C	-0.212509000	2.438377000	-0.998983000
C	0.565904000	2.092940000	-2.108495000
C	0.281788000	3.295906000	-0.014455000
C	1.858382000	2.609688000	-2.226183000
H	0.157021000	1.419643000	-2.863980000
C	1.573647000	3.810568000	-0.141654000
H	-0.320690000	3.515465000	0.867346000
C	2.362607000	3.469618000	-1.244009000
H	2.472781000	2.338277000	-3.087870000
H	1.972364000	4.460741000	0.639774000
H	3.377043000	3.865220000	-1.333480000
H	1.633611000	-3.003872000	0.582785000
C	3.872383000	-1.335093000	0.117907000
C	4.221710000	-2.529175000	-0.557376000
C	4.923981000	-0.462167000	0.490883000
C	5.555299000	-2.837005000	-0.837273000
C	6.249524000	-0.783075000	0.195849000
C	6.585186000	-1.969160000	-0.466540000

H	3.450570000	-3.229921000	-0.875492000
H	4.682152000	0.458663000	1.011571000
H	5.783993000	-3.770954000	-1.358228000
H	7.036532000	-0.086099000	0.498199000
H	7.627303000	-2.210699000	-0.689009000

INT1: E= −1523.134310

Pd	-0.301884000	-0.482651000	-0.622193000
C	-1.813082000	-2.774706000	-1.674108000
C	-1.850564000	-1.759822000	-0.705283000
C	-2.984781000	-1.681952000	0.141261000
C	-4.061556000	-2.569819000	-0.045505000
C	-4.015615000	-3.540560000	-1.051276000
C	-2.890941000	-3.650216000	-1.871735000
H	-0.929352000	-2.876654000	-2.314787000
H	-2.848111000	-4.410409000	-2.656572000
H	-4.862419000	-4.219966000	-1.180030000
H	-4.937959000	-2.521073000	0.602312000
N	-3.059205000	-0.687042000	1.149454000
C	-4.369124000	-0.212772000	1.578439000
H	-4.861221000	-0.927428000	2.260107000
H	-4.235284000	0.735355000	2.109556000
H	-5.019595000	-0.058747000	0.704723000
C	-1.937010000	-0.116005000	1.738662000
O	-2.047322000	0.830935000	2.515869000
C	-0.620291000	-0.760473000	1.405279000
C	0.603540000	-0.317241000	2.138120000
O	1.457493000	-1.087569000	2.535529000
O	0.727273000	1.014669000	2.278640000
C	1.917950000	1.489764000	2.902384000
H	1.818417000	2.580716000	2.957339000
H	2.034599000	1.064412000	3.910438000
H	2.803169000	1.226575000	2.304373000
H	-0.684061000	-1.849228000	1.528796000
C	1.340540000	0.870591000	-0.642671000
C	3.448217000	1.734185000	-0.532213000
C	2.585857000	2.777947000	-0.643253000
H	4.531764000	1.705091000	-0.467620000
H	2.760418000	3.849422000	-0.674769000
N	1.308274000	2.231532000	-0.709079000
N	2.670664000	0.578699000	-0.541037000
C	3.206106000	-0.745342000	-0.463172000
C	2.809187000	-1.713610000	-1.392921000
C	4.130129000	-1.059014000	0.539043000
C	3.325291000	-3.008766000	-1.301625000
H	2.112236000	-1.444550000	-2.188153000
C	4.652642000	-2.352209000	0.614273000
H	4.419521000	-0.301731000	1.269159000
C	4.247229000	-3.330666000	-0.299636000
H	3.012782000	-3.765139000	-2.025291000
H	5.368596000	-2.598552000	1.401670000
H	4.651423000	-4.343449000	-0.232657000
C	0.110737000	3.012377000	-0.826310000
C	-0.893114000	2.895699000	0.141425000
C	-0.033109000	3.884939000	-1.910693000
C	-2.063910000	3.645621000	0.000962000
H	-0.753598000	2.234527000	0.995209000
C	-1.202712000	4.641635000	-2.032524000
H	0.760156000	3.958230000	-2.657928000
C	-2.222198000	4.518069000	-1.081778000

H	-2.850399000	3.546290000	0.752878000
H	-1.320102000	5.321234000	-2.879939000
H	-3.138520000	5.104499000	-1.184484000

TS2: E= -1523.105339

Pd	0.055021000	-0.505817000	-0.264021000
C	-0.471617000	-3.166144000	-0.989055000
C	-1.172961000	-2.107140000	-0.389266000
C	-2.568336000	-1.984552000	-0.599141000
C	-3.208211000	-2.783270000	-1.553545000
C	-2.467321000	-3.758143000	-2.237213000
C	-1.116013000	-3.967935000	-1.944186000
H	0.579215000	-3.339956000	-0.743011000
H	-0.556966000	-4.753575000	-2.458626000
H	-2.966879000	-4.381903000	-2.982600000
H	-4.276663000	-2.672798000	-1.744645000
N	-3.225345000	-1.096784000	0.260122000
C	-4.596528000	-0.678893000	0.042304000
H	-5.289401000	-1.533663000	0.112053000
H	-4.856297000	0.052794000	0.815518000
H	-4.702853000	-0.216416000	-0.951074000
C	-2.520373000	-0.698065000	1.384383000
O	-2.972123000	0.075966000	2.213562000
C	-1.165620000	-1.374811000	1.444026000
C	-0.071491000	-0.827062000	2.310098000
O	0.858011000	-1.509966000	2.715680000
O	-0.232738000	0.461655000	2.679953000
C	0.870925000	1.086436000	3.330701000
H	0.510898000	2.064668000	3.672762000
H	1.225001000	0.488067000	4.182294000
H	1.700774000	1.225731000	2.618145000
H	-1.290423000	-2.425156000	1.742237000
C	1.249723000	1.207390000	-0.383320000
C	3.005346000	2.663395000	-0.528211000
C	1.865814000	3.396927000	-0.603327000
H	4.048485000	2.965036000	-0.530563000
H	1.712413000	4.468783000	-0.686927000
N	0.807810000	2.498002000	-0.509298000
N	2.613445000	1.333513000	-0.391365000
C	3.537148000	0.241497000	-0.345095000
C	4.573574000	0.187327000	-1.285909000
C	3.410755000	-0.755172000	0.630389000
C	5.487006000	-0.870145000	-1.250363000
H	4.653089000	0.961633000	-2.051774000
C	4.322654000	-1.814304000	0.648742000
H	2.613797000	-0.708998000	1.371792000
C	5.361588000	-1.875989000	-0.286482000
H	6.292291000	-0.911117000	-1.987667000
H	4.220765000	-2.591701000	1.409803000
H	6.072741000	-2.705199000	-0.263699000
C	-0.569630000	2.879307000	-0.609795000
C	-1.491966000	2.433567000	0.344029000
C	-0.981685000	3.687103000	-1.676398000
C	-2.838960000	2.778964000	0.210040000
H	-1.161355000	1.818430000	1.179469000
C	-2.329482000	4.042356000	-1.791523000
H	-0.254633000	4.017805000	-2.421213000
C	-3.261732000	3.582767000	-0.854641000
H	-3.549478000	2.407180000	0.951097000

H	-2.651677000	4.668907000	-2.626742000
H	-4.316043000	3.852201000	-0.954873000

2b: E= -706.559710

C	1.032857000	-1.951479000	-0.646200000
C	0.603379000	-0.634757000	-0.560170000
C	1.421860000	0.343796000	0.032293000
C	2.679147000	0.031649000	0.544709000
C	3.105178000	-1.303798000	0.452937000
C	2.298534000	-2.285590000	-0.132087000
H	0.397273000	-2.714028000	-1.103254000
H	2.652736000	-3.317146000	-0.190900000
H	4.086955000	-1.576262000	0.848258000
H	3.311493000	0.793981000	1.002441000
N	0.777882000	1.588271000	0.005628000
C	1.332023000	2.812305000	0.541778000
H	2.278711000	3.064432000	0.038030000
H	0.604699000	3.616353000	0.373330000
H	1.521878000	2.713299000	1.622714000
C	-0.470255000	1.501081000	-0.573915000
O	-1.259027000	2.413827000	-0.715072000
C	-0.676281000	0.032482000	-1.006963000
C	-1.923246000	-0.568487000	-0.373644000
O	-2.781752000	-1.167600000	-0.974105000
O	-1.928872000	-0.368405000	0.949012000
C	-3.036205000	-0.909663000	1.682039000
H	-2.868100000	-0.639214000	2.730879000
H	-3.983272000	-0.480527000	1.322867000
H	-3.073777000	-2.003533000	1.570010000
H	-0.818499000	-0.003057000	-2.097111000

INT0-cis: E= -1680.405266

Pd	0.583468000	-0.130039000	-0.744971000
C	-0.196578000	1.631509000	-0.331538000
N	-2.389190000	0.805731000	-1.157112000
C	-1.656609000	1.727782000	-0.374379000
O	-2.262711000	2.618701000	0.240163000
C	-1.706393000	-0.099719000	-2.050919000
H	-0.689951000	0.335041000	-2.269698000
C	0.625240000	2.621837000	0.304502000
O	1.695848000	2.436943000	0.900068000
O	0.168297000	3.905940000	0.154042000
C	0.940877000	4.920792000	0.775580000
H	0.411919000	5.866498000	0.592322000
H	1.037039000	4.754614000	1.861285000
H	1.957892000	4.978180000	0.352625000
C	-3.881025000	0.977070000	-1.306866000
C	-4.567376000	0.903035000	0.072318000
H	-4.201010000	1.690368000	0.737118000
H	-5.655782000	1.017745000	-0.052998000
H	-4.378843000	-0.072982000	0.544744000
C	-4.182314000	2.318845000	-2.003726000
H	-3.693209000	2.350717000	-2.991140000
H	-5.267250000	2.435098000	-2.157482000
H	-3.814468000	3.157006000	-1.400515000
C	-4.487931000	-0.151670000	-2.165941000
H	-5.580825000	-0.027645000	-2.169932000

H	-4.159565000	-0.118262000	-3.214720000
H	-4.266100000	-1.148529000	-1.760655000
C	1.611501000	-0.388487000	0.935627000
C	3.355035000	-0.802683000	2.323859000
C	2.217192000	-0.898110000	3.059119000
H	4.399405000	-0.907417000	2.602251000
H	2.060318000	-1.107857000	4.112940000
N	2.966535000	-0.492708000	1.025501000
N	1.156402000	-0.645109000	2.193681000
C	3.881199000	-0.316876000	-0.064775000
C	3.908992000	0.900451000	-0.755588000
C	4.736795000	-1.367384000	-0.412677000
C	4.800730000	1.051767000	-1.821124000
H	3.241131000	1.700703000	-0.430068000
C	5.631514000	-1.199069000	-1.474022000
H	4.692174000	-2.309453000	0.138234000
C	5.660575000	0.007335000	-2.181998000
H	4.829505000	1.997678000	-2.367449000
H	6.300503000	-2.016923000	-1.751878000
H	6.356724000	0.134719000	-3.014599000
C	-0.219985000	-0.731668000	2.589249000
C	-0.725068000	-1.966181000	3.010959000
C	-1.036059000	0.401729000	2.551901000
C	-2.067704000	-2.068188000	3.384415000
H	-0.072824000	-2.841806000	3.022933000
C	-2.381376000	0.286668000	2.909083000
H	-0.628105000	1.353834000	2.222838000
C	-2.899821000	-0.944693000	3.323410000
H	-2.467454000	-3.032618000	3.706383000
H	-3.022058000	1.167584000	2.850927000
H	-3.953819000	-1.029478000	3.598456000
H	-2.150578000	-0.065004000	-3.057855000
C	-1.599946000	-1.565388000	-1.629942000
C	-2.017097000	-2.011646000	-0.366001000
C	-1.084943000	-2.501187000	-2.544840000
C	-1.916675000	-3.363046000	-0.024426000
C	-0.976353000	-3.851280000	-2.198483000
C	-1.395616000	-4.287672000	-0.936272000
H	-2.399615000	-1.286772000	0.353680000
H	-0.767227000	-2.166012000	-3.536998000
H	-2.240483000	-3.688929000	0.966135000
H	-0.571169000	-4.565760000	-2.919771000
H	-1.318191000	-5.343851000	-0.666174000

INT0-trans: E= −1680.402314

Pd	-0.409920000	-0.449198000	-0.646509000
C	0.270946000	0.288741000	1.077287000
N	2.311389000	0.453960000	-0.255911000
C	1.590137000	0.857950000	0.909583000
O	2.107355000	1.656065000	1.700606000
C	2.174816000	-0.913979000	-0.735503000
H	2.074784000	-0.970198000	-1.828855000
H	1.243107000	-1.356226000	-0.282483000
C	-0.499996000	0.421232000	2.266774000
O	-0.192324000	0.986132000	3.317572000
O	-1.752563000	-0.182607000	2.185287000
C	-2.612069000	0.001699000	3.296373000
H	-2.166436000	-0.382191000	4.228099000
H	-2.855968000	1.066401000	3.450005000
H	-3.532506000	-0.553672000	3.066945000

C	3.439482000	1.293820000	-0.770526000
C	2.963156000	2.755634000	-0.880677000
H	2.718762000	3.175001000	0.100650000
H	3.753109000	3.370021000	-1.340612000
H	2.062942000	2.819766000	-1.510334000
C	4.688196000	1.210012000	0.132848000
H	5.099157000	0.190511000	0.145839000
H	5.472209000	1.888177000	-0.242576000
H	4.424118000	1.498280000	1.157498000
C	3.822674000	0.834429000	-2.190462000
H	4.596048000	1.510723000	-2.584044000
H	4.241716000	-0.182029000	-2.208401000
H	2.961241000	0.875006000	-2.875759000
C	-2.206353000	0.372435000	-0.434458000
C	-4.436912000	0.628496000	-0.137832000
C	-3.872629000	1.863463000	-0.083659000
H	-5.468369000	0.306546000	-0.030470000
H	-4.310442000	2.846163000	0.063438000
N	-3.402795000	-0.274296000	-0.359929000
N	-2.506823000	1.688288000	-0.276995000
C	-3.592082000	-1.691585000	-0.462976000
C	-2.898717000	-2.554110000	0.394943000
C	-4.485088000	-2.189630000	-1.418264000
C	-3.095970000	-3.932840000	0.275708000
H	-2.231914000	-2.127433000	1.144647000
C	-4.681804000	-3.570017000	-1.520236000
H	-5.011241000	-1.499915000	-2.081800000
C	-3.984355000	-4.442877000	-0.678053000
H	-2.557774000	-4.611483000	0.941781000
H	-5.375908000	-3.962800000	-2.266873000
H	-4.135846000	-5.521610000	-0.763145000
C	-1.577578000	2.782867000	-0.378539000
C	-1.316988000	3.325842000	-1.640393000
C	-0.969266000	3.299293000	0.768115000
C	-0.437875000	4.406554000	-1.755410000
H	-1.802089000	2.898686000	-2.520483000
C	-0.091819000	4.379310000	0.642758000
H	-1.151483000	2.848864000	1.743328000
C	0.172170000	4.934796000	-0.612965000
H	-0.227703000	4.831955000	-2.739447000
H	0.402566000	4.772212000	1.533448000
H	0.866636000	5.773312000	-0.703584000
C	3.237892000	-1.898666000	-0.268013000
C	3.632967000	-1.914951000	1.079152000
C	3.799768000	-2.826332000	-1.154723000
C	4.578286000	-2.839998000	1.527472000
H	3.207622000	-1.184394000	1.770815000
C	4.748710000	-3.753219000	-0.707962000
H	3.498678000	-2.819047000	-2.206554000
C	5.140130000	-3.761666000	0.634480000
H	4.881062000	-2.841102000	2.577762000
H	5.186133000	-4.466361000	-1.411525000
H	5.883007000	-4.482657000	0.985170000

TS1-cis: E= -1680.385852

Pd	-0.373548000	0.481153000	-0.326986000
C	0.568158000	-1.137525000	-1.172055000
N	2.503833000	0.274825000	-1.140672000
C	2.021629000	-1.059448000	-1.078904000
O	2.787919000	-2.020344000	-0.996948000

C	1.480610000	1.254012000	-1.394854000
H	0.566607000	0.303379000	-1.790937000
C	-0.233150000	-2.290947000	-1.441213000
O	-1.462584000	-2.318666000	-1.554440000
O	0.477341000	-3.455477000	-1.568908000
C	-0.277690000	-4.611900000	-1.906059000
H	0.444917000	-5.435205000	-1.989488000
H	-1.023894000	-4.848954000	-1.130465000
H	-0.810051000	-4.484820000	-2.862871000
C	3.939490000	0.558936000	-1.458000000
C	4.828892000	0.015619000	-0.323036000
H	4.699549000	-1.066230000	-0.211587000
H	5.886960000	0.231936000	-0.541734000
H	4.568479000	0.500797000	0.630787000
C	4.314332000	-0.084254000	-2.807614000
H	3.671520000	0.311851000	-3.610904000
H	5.361196000	0.142089000	-3.065717000
H	4.189154000	-1.173576000	-2.762905000
C	4.173638000	2.077495000	-1.553608000
H	5.240640000	2.253687000	-1.756077000
H	3.604568000	2.543082000	-2.371487000
H	3.917397000	2.590915000	-0.616672000
C	-1.693868000	-0.478033000	0.874585000
C	-3.532617000	-1.314364000	1.906205000
C	-2.446899000	-1.742396000	2.603065000
H	-4.592556000	-1.503341000	2.047077000
H	-2.362932000	-2.370231000	3.485161000
N	-3.053407000	-0.540006000	0.852281000
N	-1.331775000	-1.220102000	1.957663000
C	-3.888833000	0.131733000	-0.098186000
C	-3.645355000	-0.020814000	-1.468526000
C	-4.939979000	0.932496000	0.363833000
C	-4.461473000	0.656453000	-2.379588000
H	-2.838151000	-0.683943000	-1.787737000
C	-5.755933000	1.593422000	-0.559034000
H	-5.107622000	1.047258000	1.436948000
C	-5.514767000	1.461845000	-1.930901000
H	-4.278617000	0.541921000	-3.450960000
H	-6.575811000	2.221118000	-0.201889000
H	-6.150328000	1.984355000	-2.650003000
C	0.023967000	-1.331677000	2.425765000
C	0.462070000	-0.462358000	3.430254000
C	0.899995000	-2.244555000	1.833360000
C	1.797818000	-0.501382000	3.839050000
H	-0.238102000	0.253495000	3.865658000
C	2.236879000	-2.269447000	2.239190000
H	0.556730000	-2.897532000	1.032705000
C	2.687135000	-1.399616000	3.238010000
H	2.146172000	0.182439000	4.616684000
H	2.928368000	-2.953972000	1.745940000
H	3.735874000	-1.416852000	3.544414000
H	1.526413000	1.712721000	-2.397462000
C	1.220256000	2.293962000	-0.342967000
C	1.696658000	2.143683000	0.981680000
C	0.469689000	3.448808000	-0.661295000
C	1.432920000	3.117796000	1.944264000
C	0.206569000	4.417400000	0.311769000
C	0.689818000	4.259523000	1.615260000
H	2.275891000	1.253834000	1.233637000
H	0.104788000	3.584845000	-1.683078000
H	1.808775000	2.981133000	2.961373000

H	-0.372758000	5.305184000	0.045823000
H	0.488330000	5.021676000	2.371904000

TS1–trans: E= –1680.383476

Pd	0.290727000	0.260294000	-0.638509000
C	-0.544478000	-0.377312000	1.142036000
N	-2.643846000	-0.110220000	0.054535000
C	-1.901838000	-0.895086000	0.967112000
O	-2.392041000	-1.857421000	1.556023000
C	-1.895266000	0.986022000	-0.509727000
H	-1.651396000	0.850926000	-1.583342000
H	-0.952564000	0.888327000	0.473398000
C	0.285125000	-0.661784000	2.270973000
O	0.028196000	-1.403507000	3.215668000
O	1.492058000	0.007415000	2.242632000
C	2.407553000	-0.295439000	3.284568000
H	1.981262000	-0.079578000	4.276991000
H	2.705052000	-1.356578000	3.260357000
H	3.288945000	0.336754000	3.110084000
C	-3.927006000	-0.598228000	-0.537586000
C	-3.677812000	-1.958756000	-1.219877000
H	-3.319209000	-2.700460000	-0.495612000
H	-4.606150000	-2.332713000	-1.680336000
H	-2.917564000	-1.856609000	-2.011161000
C	-4.997138000	-0.728186000	0.563105000
H	-5.193186000	0.254110000	1.020911000
H	-5.940323000	-1.100759000	0.131507000
H	-4.660770000	-1.420004000	1.343696000
C	-4.442096000	0.391954000	-1.596238000
H	-5.384081000	0.002102000	-2.010595000
H	-4.645426000	1.383119000	-1.168325000
H	-3.738449000	0.512678000	-2.433106000
C	2.049846000	-0.750632000	-0.490223000
C	4.197755000	-1.347789000	-0.061083000
C	3.454304000	-2.485428000	-0.074205000
H	5.256216000	-1.187025000	0.120366000
H	3.730778000	-3.524943000	0.074817000
N	3.323565000	-0.295525000	-0.321130000
N	2.147000000	-2.100091000	-0.346402000
C	3.730852000	1.075814000	-0.394319000
C	3.069858000	2.047569000	0.367325000
C	4.801696000	1.423419000	-1.226531000
C	3.479980000	3.380831000	0.274268000
H	2.260912000	1.742230000	1.031158000
C	5.210808000	2.757936000	-1.300789000
H	5.301294000	0.654722000	-1.820068000
C	4.547861000	3.739400000	-0.555914000
H	2.966172000	4.141760000	0.866664000
H	6.044686000	3.030748000	-1.951820000
H	4.865996000	4.782690000	-0.620088000
C	1.049382000	-3.013322000	-0.521448000
C	0.647932000	-3.345241000	-1.819904000
C	0.385318000	-3.529963000	0.592526000
C	-0.434958000	-4.209093000	-2.003088000
H	1.180406000	-2.919382000	-2.672784000
C	-0.696610000	-4.392753000	0.399431000
H	0.673389000	-3.223257000	1.598185000
C	-1.106706000	-4.733506000	-0.892762000

H	-0.756798000	-4.468063000	-3.014533000
H	-1.237773000	-4.773079000	1.267574000
H	-1.960692000	-5.399587000	-1.037018000
C	-2.367272000	2.380858000	-0.247610000
C	-3.099756000	2.687211000	0.912308000
C	-2.022142000	3.421792000	-1.126453000
C	-3.488719000	4.002339000	1.178226000
H	-3.365013000	1.879871000	1.598058000
C	-2.406977000	4.737555000	-0.857465000
H	-1.453104000	3.193819000	-2.032806000
C	-3.144456000	5.033379000	0.295622000
H	-4.062371000	4.225081000	2.081852000
H	-2.136448000	5.535462000	-1.554079000
H	-3.450176000	6.061702000	0.504188000

INT1-cis: E= -1680.471183

Pd	-0.139027000	0.474328000	0.308950000
C	0.581342000	0.566272000	-1.614113000
N	2.675760000	1.058244000	-0.493577000
C	2.002209000	1.106494000	-1.677808000
O	2.451413000	1.520345000	-2.744899000
C	1.853232000	0.839475000	0.682725000
H	-0.076605000	1.199750000	-2.223432000
C	0.526387000	-0.842823000	-2.092979000
O	1.431461000	-1.656231000	-2.028076000
O	-0.668649000	-1.150229000	-2.650451000
C	-0.792644000	-2.475487000	-3.168464000
H	-0.106218000	-2.626769000	-4.016655000
H	-0.570239000	-3.222893000	-2.394861000
H	-1.832420000	-2.580249000	-3.500681000
C	4.089395000	1.556987000	-0.385320000
C	4.987902000	0.769243000	-1.356839000
H	4.656789000	0.896017000	-2.393862000
H	6.028021000	1.121465000	-1.271033000
H	4.965230000	-0.303125000	-1.107806000
C	4.111604000	3.064542000	-0.707266000
H	3.477731000	3.615678000	0.006487000
H	5.137682000	3.457068000	-0.627241000
H	3.741915000	3.250417000	-1.723547000
C	4.633343000	1.351698000	1.038285000
H	5.659090000	1.748438000	1.076002000
H	4.041498000	1.885105000	1.795284000
H	4.668835000	0.289590000	1.314260000
C	-2.206805000	0.075126000	0.172140000
C	-4.461113000	0.418590000	0.261752000
C	-4.258653000	-0.921263000	0.200845000
H	-5.376159000	1.001344000	0.311514000
H	-4.967378000	-1.743086000	0.215323000
N	-3.202145000	1.008226000	0.238420000
N	-2.879088000	-1.117190000	0.147420000
C	-2.990524000	2.422839000	0.312804000
C	-2.238679000	3.072657000	-0.672897000
C	-3.551081000	3.143450000	1.373098000
C	-2.035125000	4.451719000	-0.583372000
H	-1.820905000	2.496469000	-1.497708000
C	-3.351204000	4.525325000	1.449042000
H	-4.128426000	2.620345000	2.138404000
C	-2.589522000	5.180161000	0.475679000
H	-1.446458000	4.959324000	-1.351014000
H	-3.785686000	5.088281000	2.278391000

H	-2.429930000	6.259041000	0.540291000
C	-2.280719000	-2.415198000	0.068632000
C	-1.100561000	-2.708726000	0.764212000
C	-2.905445000	-3.405748000	-0.701647000
C	-0.538537000	-3.983630000	0.663007000
H	-0.619305000	-1.950771000	1.383337000
C	-2.349672000	-4.684923000	-0.775542000
H	-3.809106000	-3.172296000	-1.266936000
C	-1.160170000	-4.976585000	-0.100795000
H	0.388300000	-4.193667000	1.199348000
H	-2.840159000	-5.448745000	-1.383367000
H	-0.719025000	-5.973605000	-0.169555000
H	1.777406000	1.759402000	1.290988000
C	2.093520000	-0.365975000	1.507959000
C	2.629441000	-1.555572000	0.962688000
C	1.681231000	-0.381291000	2.861376000
C	2.741377000	-2.705804000	1.740169000
C	1.780879000	-1.544241000	3.631359000
C	2.313777000	-2.712631000	3.077249000
H	2.917344000	-1.563724000	-0.088902000
H	1.288060000	0.536597000	3.309231000
H	3.156755000	-3.615641000	1.297844000
H	1.452031000	-1.532643000	4.674110000
H	2.399978000	-3.620335000	3.679770000

INT1-trans: E= -1680.469363

Pd	0.007969000	-0.062700000	-0.547169000
C	-0.182280000	-0.714987000	1.393039000
N	-2.043095000	-1.961379000	0.504952000
C	-1.127865000	-1.894186000	1.520204000
O	-1.087158000	-2.641134000	2.495018000
C	-1.696034000	-1.223433000	-0.696916000
H	-1.423100000	-1.904942000	-1.521869000
H	-0.659625000	0.160578000	1.859685000
C	1.179316000	-0.938829000	1.943905000
O	1.870021000	-1.927459000	1.802658000
O	1.628360000	0.157439000	2.613114000
C	2.980537000	0.121617000	3.064167000
H	3.154687000	-0.735726000	3.731604000
H	3.673488000	0.048992000	2.212518000
H	3.151793000	1.062975000	3.600936000
C	-3.109541000	-3.019240000	0.491930000
C	-2.437500000	-4.402731000	0.390582000
H	-1.774748000	-4.574887000	1.248176000
H	-3.199655000	-5.197645000	0.364576000
H	-1.841136000	-4.469672000	-0.533991000
C	-3.962576000	-2.911622000	1.770035000
H	-4.442003000	-1.921269000	1.823477000
H	-4.755278000	-3.676139000	1.754103000
H	-3.351924000	-3.056695000	2.668402000
C	-4.042027000	-2.833204000	-0.716636000
H	-4.798761000	-3.631790000	-0.694950000
H	-4.564226000	-1.867441000	-0.685925000
H	-3.507925000	-2.905538000	-1.674287000
C	1.734167000	1.113154000	-0.395473000
C	3.079820000	2.842121000	0.225544000
C	3.887200000	1.830351000	-0.189846000
H	3.311553000	3.837285000	0.593750000
H	4.968089000	1.769804000	-0.273073000
N	1.775180000	2.385071000	0.084952000

N	3.048698000	0.784001000	-0.572024000
C	0.593790000	3.125786000	0.428609000
C	0.037692000	2.974641000	1.702146000
C	-0.004629000	3.950650000	-0.528151000
C	-1.139921000	3.658396000	2.016775000
H	0.519797000	2.302045000	2.413055000
C	-1.183045000	4.630084000	-0.205250000
H	0.445584000	4.042307000	-1.518694000
C	-1.751338000	4.482065000	1.064753000
H	-1.586377000	3.538519000	3.006685000
H	-1.662677000	5.267565000	-0.951382000
H	-2.676872000	5.007495000	1.312149000
C	3.504692000	-0.472711000	-1.075892000
C	2.883273000	-1.043608000	-2.193868000
C	4.571412000	-1.126819000	-0.449050000
C	3.318222000	-2.284268000	-2.666521000
H	2.078719000	-0.507444000	-2.699400000
C	5.009795000	-2.359884000	-0.938196000
H	5.045434000	-0.685255000	0.429172000
C	4.380926000	-2.945572000	-2.041615000
H	2.829502000	-2.729078000	-3.536521000
H	5.838311000	-2.870797000	-0.442325000
H	4.719939000	-3.914434000	-2.415551000
C	-2.580847000	-0.117711000	-1.126367000
C	-3.367571000	0.613643000	-0.203390000
C	-2.572042000	0.314633000	-2.473069000
C	-4.092262000	1.731170000	-0.608199000
H	-3.384659000	0.291270000	0.840162000
C	-3.295576000	1.443303000	-2.872890000
H	-1.994116000	-0.252056000	-3.209709000
C	-4.058018000	2.158954000	-1.945251000
H	-4.686753000	2.283928000	0.124408000
H	-3.269643000	1.758227000	-3.919751000
H	-4.625865000	3.038819000	-2.257796000

TS2-cis: E= -1680.424287

Pd	0.165904000	0.143553000	-0.421642000
C	-1.293420000	0.970988000	1.359426000
N	-3.284966000	0.539093000	0.492747000
C	-2.757576000	0.770235000	1.720211000
O	-3.293360000	0.843088000	2.810101000
C	-2.136432000	0.692475000	-0.380972000
H	-1.010305000	2.028368000	1.427787000
C	-0.366324000	0.081308000	2.121087000
O	-0.574661000	-1.094667000	2.372935000
O	0.670001000	0.771222000	2.669173000
C	1.671103000	-0.011866000	3.314879000
H	1.245919000	-0.594561000	4.145681000
H	2.134501000	-0.706011000	2.597193000
H	2.421104000	0.695419000	3.690341000
C	-4.726417000	0.572763000	0.124119000
C	-5.442275000	-0.556711000	0.883439000
H	-5.299712000	-0.442484000	1.966699000
H	-6.520850000	-0.534634000	0.663045000
H	-5.044727000	-1.536919000	0.578372000
C	-5.297000000	1.945621000	0.527649000
H	-4.777738000	2.753470000	-0.012377000
H	-6.368846000	2.000577000	0.282144000
H	-5.176742000	2.112329000	1.607476000
C	-4.888502000	0.370435000	-1.388208000

H	-5.956439000	0.447809000	-1.641834000
H	-4.349268000	1.138165000	-1.963260000
H	-4.529958000	-0.617132000	-1.708532000
C	2.193927000	0.099020000	-0.214625000
C	4.391040000	0.730980000	-0.102911000
C	4.351100000	-0.613886000	0.067066000
H	5.225597000	1.425996000	-0.112847000
H	5.147530000	-1.336930000	0.214874000
N	3.072218000	1.149408000	-0.265786000
N	3.009060000	-0.986684000	-0.008864000
C	2.691028000	2.516991000	-0.439284000
C	1.794202000	3.105880000	0.460872000
C	3.228667000	3.254326000	-1.499271000
C	1.423648000	4.440979000	0.281427000
H	1.404451000	2.512545000	1.288682000
C	2.858518000	4.593113000	-1.664771000
H	3.921346000	2.775963000	-2.195080000
C	1.952202000	5.186297000	-0.779784000
H	0.723326000	4.903555000	0.981285000
H	3.274318000	5.169600000	-2.494569000
H	1.660368000	6.230661000	-0.914408000
C	2.558549000	-2.334264000	0.147699000
C	1.634230000	-2.885420000	-0.748463000
C	3.058101000	-3.099629000	1.209439000
C	1.206797000	-4.202979000	-0.568917000
H	1.249192000	-2.286698000	-1.574757000
C	2.630147000	-4.419744000	1.375308000
H	3.767235000	-2.657681000	1.912437000
C	1.700769000	-4.973619000	0.489229000
H	0.481829000	-4.626179000	-1.267003000
H	3.017864000	-5.011012000	2.208244000
H	1.361949000	-6.003696000	0.622917000
C	-1.830351000	-0.419561000	-1.307350000
C	-1.224289000	-0.155254000	-2.568668000
C	-2.112829000	-1.772189000	-0.965755000
C	-0.935438000	-1.212301000	-3.451115000
H	-1.082568000	0.877837000	-2.896040000
C	-1.823958000	-2.799991000	-1.852347000
H	-2.559576000	-1.984991000	0.006816000
C	-1.233758000	-2.527635000	-3.101769000
H	-0.483977000	-0.988729000	-4.420943000
H	-2.052608000	-3.831412000	-1.572721000
H	-1.012373000	-3.344626000	-3.792679000
H	-2.085200000	1.679954000	-0.861580000

TS2-trans: E= -1680.423021

Pd	0.324920000	-0.065020000	-0.372178000
C	-0.984991000	-0.996544000	1.359781000
N	-2.374467000	-2.083177000	0.062619000
C	-1.834261000	-2.262156000	1.291039000
O	-1.981057000	-3.153980000	2.104943000
C	-1.781855000	-0.825933000	-0.364692000
H	-1.200179000	-0.958641000	-1.315217000
H	-1.466652000	-0.196248000	1.933778000
C	0.429582000	-1.214974000	1.814905000
O	1.071719000	-2.242891000	1.665110000
O	0.848020000	-0.203798000	2.631924000
C	2.218912000	-0.241729000	3.019074000
H	2.471099000	-1.197277000	3.501996000
H	2.867726000	-0.107961000	2.137989000

H	2.366261000	0.590000000	3.719469000
C	-3.300389000	-2.979602000	-0.672505000
C	-2.618772000	-4.353041000	-0.813587000
H	-2.380325000	-4.767181000	0.175894000
H	-3.284090000	-5.054726000	-1.340203000
H	-1.683523000	-4.260558000	-1.387617000
C	-4.607457000	-3.107149000	0.129628000
H	-5.118157000	-2.134694000	0.197026000
H	-5.286348000	-3.821579000	-0.361278000
H	-4.397056000	-3.466179000	1.147212000
C	-3.586644000	-2.394590000	-2.061177000
H	-4.269115000	-3.070031000	-2.598383000
H	-4.063172000	-1.406182000	-1.996625000
H	-2.667293000	-2.299652000	-2.658717000
C	2.050255000	1.021337000	-0.404694000
C	3.434863000	2.829742000	-0.204350000
C	4.220312000	1.741623000	-0.407583000
H	3.687816000	3.875413000	-0.054987000
H	5.298699000	1.648037000	-0.493420000
N	2.119837000	2.374220000	-0.197317000
N	3.363429000	0.648474000	-0.531328000
C	0.980571000	3.210488000	0.032965000
C	0.102048000	2.910747000	1.082758000
C	0.750829000	4.310483000	-0.799068000
C	-1.026162000	3.709601000	1.279082000
H	0.304659000	2.052157000	1.724531000
C	-0.375713000	5.112112000	-0.585390000
H	1.441236000	4.523688000	-1.618019000
C	-1.268845000	4.808063000	0.446695000
H	-1.723711000	3.466896000	2.083884000
H	-0.561014000	5.967555000	-1.239370000
H	-2.157531000	5.424561000	0.600366000
C	3.813100000	-0.695146000	-0.716761000
C	3.207585000	-1.517671000	-1.674556000
C	4.861478000	-1.181457000	0.073773000
C	3.648600000	-2.834031000	-1.828647000
H	2.396762000	-1.121486000	-2.286607000
C	5.304158000	-2.496396000	-0.096000000
H	5.316200000	-0.539247000	0.830761000
C	4.696516000	-3.326967000	-1.042693000
H	3.172744000	-3.476093000	-2.573701000
H	6.118773000	-2.874676000	0.525999000
H	5.038222000	-4.357082000	-1.168041000
C	-2.699562000	0.356596000	-0.441746000
C	-3.741645000	0.522042000	0.488056000
C	-2.513461000	1.346553000	-1.423995000
C	-4.562734000	1.651420000	0.447279000
H	-3.911929000	-0.240730000	1.250793000
C	-3.334674000	2.474639000	-1.465700000
H	-1.713405000	1.231433000	-2.159150000
C	-4.361330000	2.633947000	-0.528775000
H	-5.364522000	1.764196000	1.181516000
H	-3.168875000	3.235528000	-2.232096000
H	-5.002548000	3.518349000	-0.559934000

cis-7a: E= -863.862392

C	0.070121000	-1.557573000	-0.772417000
N	1.539541000	-0.189118000	-0.160782000
C	1.423408000	-1.546405000	-0.043444000
O	2.159194000	-2.394092000	0.413330000

C	0.310828000	-0.004015000	-0.954835000
H	0.074947000	-2.109307000	-1.723052000
C	-1.141173000	-1.919943000	0.049629000
O	-1.135918000	-2.152607000	1.236393000
O	-2.246541000	-1.885314000	-0.702516000
C	-3.495036000	-2.056878000	-0.018057000
H	-3.528113000	-3.029514000	0.494464000
H	-3.633757000	-1.255863000	0.723761000
H	-4.274558000	-2.002661000	-0.786829000
C	2.724050000	0.675671000	0.018220000
C	3.232819000	0.496452000	1.458126000
H	3.481398000	-0.556589000	1.650660000
H	4.132935000	1.109470000	1.619279000
H	2.463250000	0.810105000	2.180416000
C	3.800981000	0.243310000	-0.994268000
H	3.435297000	0.366458000	-2.026170000
H	4.709588000	0.853747000	-0.875704000
H	4.067820000	-0.813242000	-0.842158000
C	2.320379000	2.135261000	-0.224426000
H	3.206025000	2.778648000	-0.113496000
H	1.921244000	2.281339000	-1.239966000
H	1.558597000	2.466563000	0.495347000
C	-0.740993000	0.926457000	-0.402589000
C	-1.624737000	1.571680000	-1.278804000
C	-0.901048000	1.103143000	0.980286000
C	-2.652083000	2.380665000	-0.784315000
H	-1.507787000	1.438354000	-2.357949000
C	-1.925763000	1.913469000	1.476414000
H	-0.218609000	0.598819000	1.667044000
C	-2.805217000	2.553643000	0.595531000
H	-3.332904000	2.879921000	-1.478421000
H	-2.038147000	2.045620000	2.555471000
H	-3.605908000	3.188198000	0.983750000
H	0.518810000	0.240224000	-2.008935000

trans-7a: E= -863.864629

C	-1.112044000	-0.616886000	0.490661000
N	-0.058166000	1.164179000	0.119926000
C	-1.161479000	0.892473000	0.867358000
O	-1.912490000	1.581259000	1.521346000
C	0.091239000	-0.207558000	-0.415555000
H	-0.181069000	-0.270054000	-1.479961000
H	-0.861239000	-1.269962000	1.336383000
C	-2.353861000	-1.080822000	-0.226055000
O	-2.627765000	-0.804065000	-1.372961000
O	-3.143742000	-1.796547000	0.579592000
C	-4.399007000	-2.225892000	0.033443000
H	-5.007320000	-1.358706000	-0.264104000
H	-4.238450000	-2.866039000	-0.846816000
H	-4.900807000	-2.789099000	0.828792000
C	0.487257000	2.461669000	-0.328241000
C	-0.580531000	3.165930000	-1.185959000
H	-1.496168000	3.331766000	-0.598850000
H	-0.209978000	4.140834000	-1.538830000
H	-0.837901000	2.552911000	-2.064064000
C	0.820747000	3.300056000	0.916617000
H	1.591350000	2.799902000	1.523504000
H	1.200966000	4.288438000	0.615807000
H	-0.075493000	3.440510000	1.537476000
C	1.753532000	2.207395000	-1.155201000

H	2.152587000	3.168906000	-1.511037000
H	2.530081000	1.708872000	-0.557776000
H	1.541498000	1.582292000	-2.036257000
C	1.406306000	-0.898735000	-0.153817000
C	2.107220000	-0.685992000	1.043648000
C	1.927612000	-1.794338000	-1.097106000
C	3.306308000	-1.357754000	1.292391000
H	1.713641000	0.021133000	1.778276000
C	3.126867000	-2.471023000	-0.848844000
H	1.392785000	-1.960061000	-2.036614000
C	3.819448000	-2.253957000	0.346368000
H	3.844742000	-1.180455000	2.226920000
H	3.523435000	-3.165012000	-1.594334000
H	4.758803000	-2.778016000	0.539926000
